# Inhibition and Rescue of Hyperglycemia‐Induced Cellular Senescence by Mitochondrial Transfer from Enucleated Mesenchymal Stem Cell‐Derived Microvesicles for Chronic Wound Healing

**DOI:** 10.1002/advs.202501612

**Published:** 2025-05-23

**Authors:** Zixuan Dong, Xiaobing Liu, Shichun Li, Xiaoling Fu

**Affiliations:** ^1^ School of Biomedical Sciences and Engineering South China University of Technology Guangzhou International Campus Guangzhou 511442 P. R. China; ^2^ National Engineering Research Center for Tissue Restoration and Reconstruction and Innovation Center for Tissue Restoration and Reconstruction Guangzhou 510006 P. R. China; ^3^ Laboratory of Biomedical Engineering of Guangdong Province South China University of Technology Guangzhou 510006 P. R. China

**Keywords:** cellular senescence, diabetic pressure sore, enucleated mesenchymal stem cells, mitochondrial transfer

## Abstract

The aberrant cellular senescence in chronic wounds presents a significant barrier to healing. Mitochondrial dysfunction is critical in initiating and maintaining cellular senescence, underscoring therapeutic potential in restoring mitochondrial function by delivering healthy mitochondria to wound cells. However, approaches for delivering mitochondria to achieve optimized wound repair remain lacking. Herein, enucleated MSCs‐derived microvesicles containing functional mitochondria (Mito@euMVs) via simple extrusion are developed. By controlling the size of microvesicles within a small micron‐scale range, the mitochondrial encapsulation efficiency is optimized. Mito@euMVs effectively delivered mitochondria into fibroblasts and HUVECs, inhibiting and rejuvenating hyperglycemia‐induced cellular senescence. To enhance the clinical applicability, soluble PVA microneedle patches for the transdermal Mito@euMVs delivery are utilized. In diabetic rats with pressure sores, the senescence‐inhibiting and ‐rescuing properties of Mito@euMVs are further validated, along with their therapeutic efficacy, demonstrating their potential for chronic wound repair. Moreover, as a versatile delivery vehicle for mitochondria, Mito@euMVs hold promising for treating mitochondrial dysfunction and aging‐related conditions.

## Graphical abstract

This study develops enucleated MSC‐derived microvesicles (Mito@euMVs) to deliver functional mitochondria for optimizing wound repair. By efficiently encapsulating mitochondria, Mito@euMVs rejuvenate hyperglycemia‐induced senescent fibroblasts and HUVECs. Using PVA microneedle patches, the therapeutic efficacy of Mito@euMVs is validated in diabetic rats with pressure sores. Mito@euMVs offer a promising strategy for chronic wound healing and mitochondrial dysfunction‐related conditions.

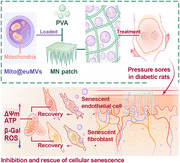

## 1 Introduction

The repair of chronic wounds remains a challenging clinical problem due to multiple factors that collectively instigate diverse pathological changes at the wound site, which disrupt and ultimately impede normal healing processes.^[^ ^1^ ^]^ Among these factors, the aberrant accumulation of senescent cells induced by stress, such as hyperglycemia, has been recognized as a crucial factor that detrimentally affects the repair of chronic wounds.^[^ ^2^ ^]^ Senescent cells (e.g., fibroblasts) are not only unable to divide but also fail to mount effective responses to various pro‐repair signals, including growth factors and cytokines. Additionally, these cells exhibit a senescence‐associated secretory phenotype (SASP), secreting a large number of factors and producing excessive reactive oxygen species (ROS), thereby exacerbating the wound microenvironment.^[^ ^3^ ^]^ Apparently, strategies that target senescence‐linked processes hold great promise for wound healing. Considerable evidence indicates that mitochondrial dysfunction is a hallmark of cellular senescence and is intimately involved in and potentially required for the induction and maintenance of the senescent state.^[^ ^4^ ^]^ For instance, dysfunctional mitochondria in senescent cells produce excessive ROS, which leads to oxidative DNA damage and the DNA damage response signaling pathway, contributing to cell cycle arrest^[^ ^5^ ^]^ Moreover, mitochondrial damage‐associated molecular patterns (DAMPs) are recognized by the innate immune system, triggering inflammasome assembly and the activation of proinflammatory cytokines, thereby stimulating the SASP.^[^ ^6^ ^]^ Undoubtedly, the causal role of mitochondria in cellular senescence opens the door for interventions to counteract their detrimental effects. However, approaches targeting mitochondria for optimized wound repair remain lacking.

The exogenous administration of mesenchymal stromal cells (MSCs) is therapeutically effective for multiple tissue injuries, including diabetic wounds.^[^ ^7^ ^]^ In addition to cell replacement and paracrine functions, MSCs have been demonstrated to rescue and revitalize injured cells by donating mitochondria through various mechanisms, such as tunneling nanotubes, extracellular vesicles, cytoplasmic fusion, and naked mitochondria ejection.^[^ ^8^ ^]^ Such intercellular mitochondrial transfer benefits cells with dysfunctional mitochondria by restoring mitochondrial function, as indicated by improved oxidative phosphorylation (OXPHOS), increased ATP production, decreased ROS, etc. Thus far, direct transfer of mitochondria from MSCs has been explored as a possible treatment approach for a variety of diseases, such as acute lung injury,^[^ ^9^ ^]^ myocardial ischemia‒reperfusion injury,^[^ ^10^ ^]^ pulmonary arterial hypertension,^[^ ^11^ ^]^ and osteoarthritis.^[^ ^12^ ^]^ However, it is not known whether mitochondrial transfer by MSCs is capable of rescuing cell senescence in chronic wounds. Besides, while the idea of transferring MSCs’ mitochondria to rescue senescent cells is intriguing, some issues remain to be addressed before their translation to the clinic. First and foremost, the amount of spontaneous intercellular mitochondria donated by MSCs is extremely limited,^[^ ^13^ ^]^ likely insufficient to effectively regulate the cellular senescence process. In addition, as with any stem cell‐based therapy, significant obstacles to direct administration of MSCs include oncogenic transformation,^[^ ^14^ ^]^ undesired differentiation,^[^ ^15^ ^]^ immunological rejection,^[^ ^16^ ^]^ and blood vessel occlusion.^[^ ^17^ ^]^ Recently, several studies have explored the transfer of isolated naked mitochondria into tissues, observing certain benefits for the repair process.^[^ ^10^, ^18^ ^]^ However, the structural integrity and function of mitochondria are likely compromised due to the high‐calcium extracellular environment.^[^ ^19^ ^]^ Thus, alternative strategies to address these challenges are highly desired.

**Scheme 1:**
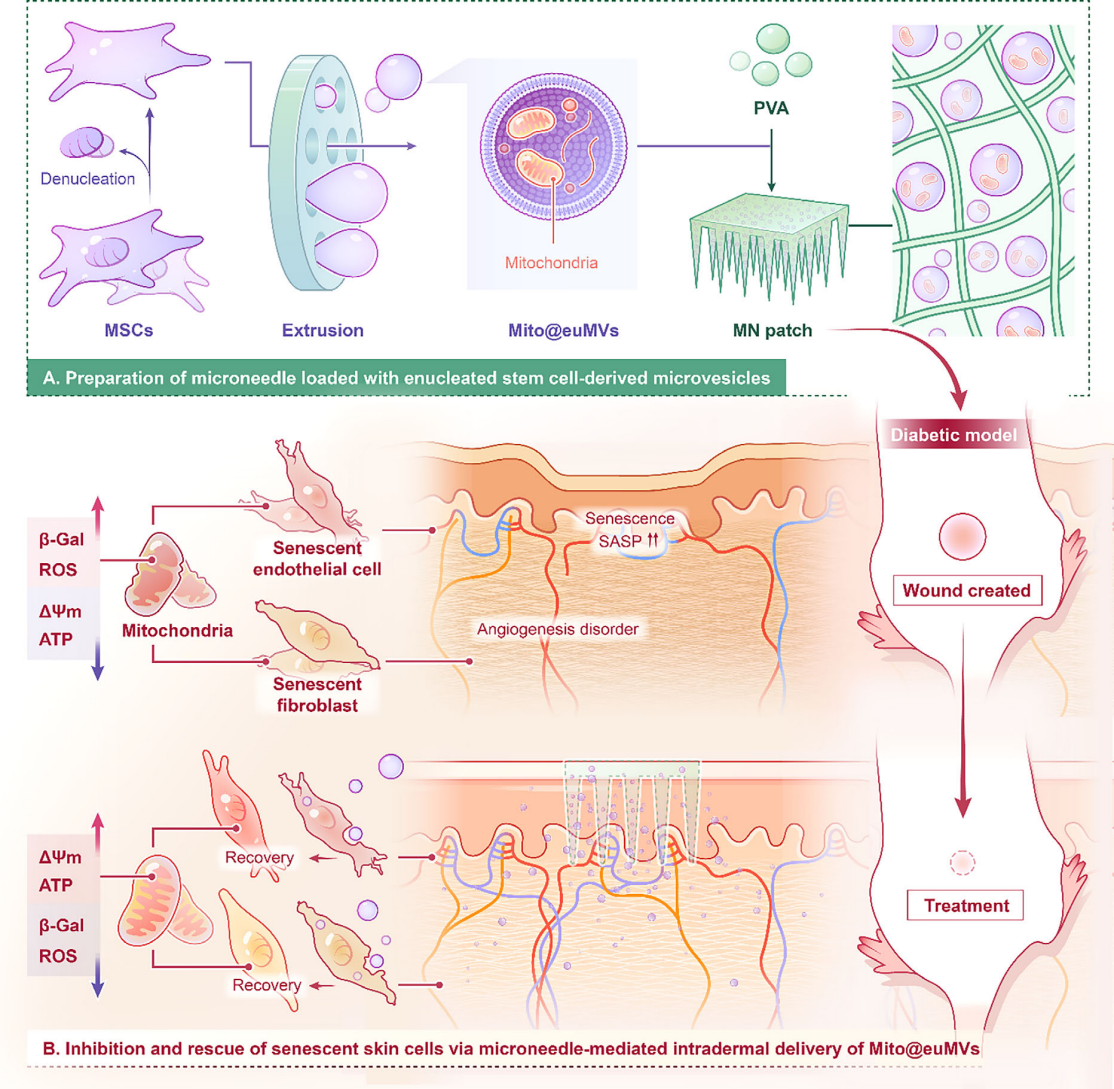
A dissolving PVA microneedle patch loaded with Mito@euMVs for the healing of diabetic pressure sore. A) A diagram showing the preparation of the microneedle patch loaded with the enucleated MSCs‐derived microvesicles containing mitochondria (Mito@euMVs). B) Inhibition and rescue of cellular senescence and improvement of mitochondrial function by Mito@euMVs in diabetic microenvironment.

To develop a novel strategy for delivering mitochondria to rescue cellular senescence and address the issues associated with transferring MSC's mitochondria, we prepared 3‐µm enucleated MSCs‐derived microvesicles containing active mitochondria (Mito@euMVs) through an extrusion approach (**Scheme** **1**). By directly extruding enucleated MSCs into MVs with controllable sizes, we maximized the rate of mitochondrial inclusion in MVs. Furthermore, the encapsulation of mitochondria within MVs shields them from the external environment, such as high Ca^2+^. To avoid the potential risks of foreign gene transfer via the nuclear genes of MSCs, MSCs’ nuclei were removed before extrusion. The ability of the obtained Mito@euMVs to encapsulate mitochondria, maintain mitochondrial activity, and deliver mitochondria into cells was evaluated. The regulatory effects of the Mito@euMVs on the senescent phenotype in a high‐glucose environment were also assessed. Moreover, a soluble PVA microneedle (MN) patch was fabricated to facilitate the transdermal delivery of the Mito@euMVs. Then, the in vivo efficacy of the Mito@euMVs was investigated in a rat model of diabetic pressure sores.

## 2 Results

### 2.1 Three‐Micrometer Microvesicles Derived from Enucleated MSCs Encapsulate Active Mitochondria

**Figure 1:**
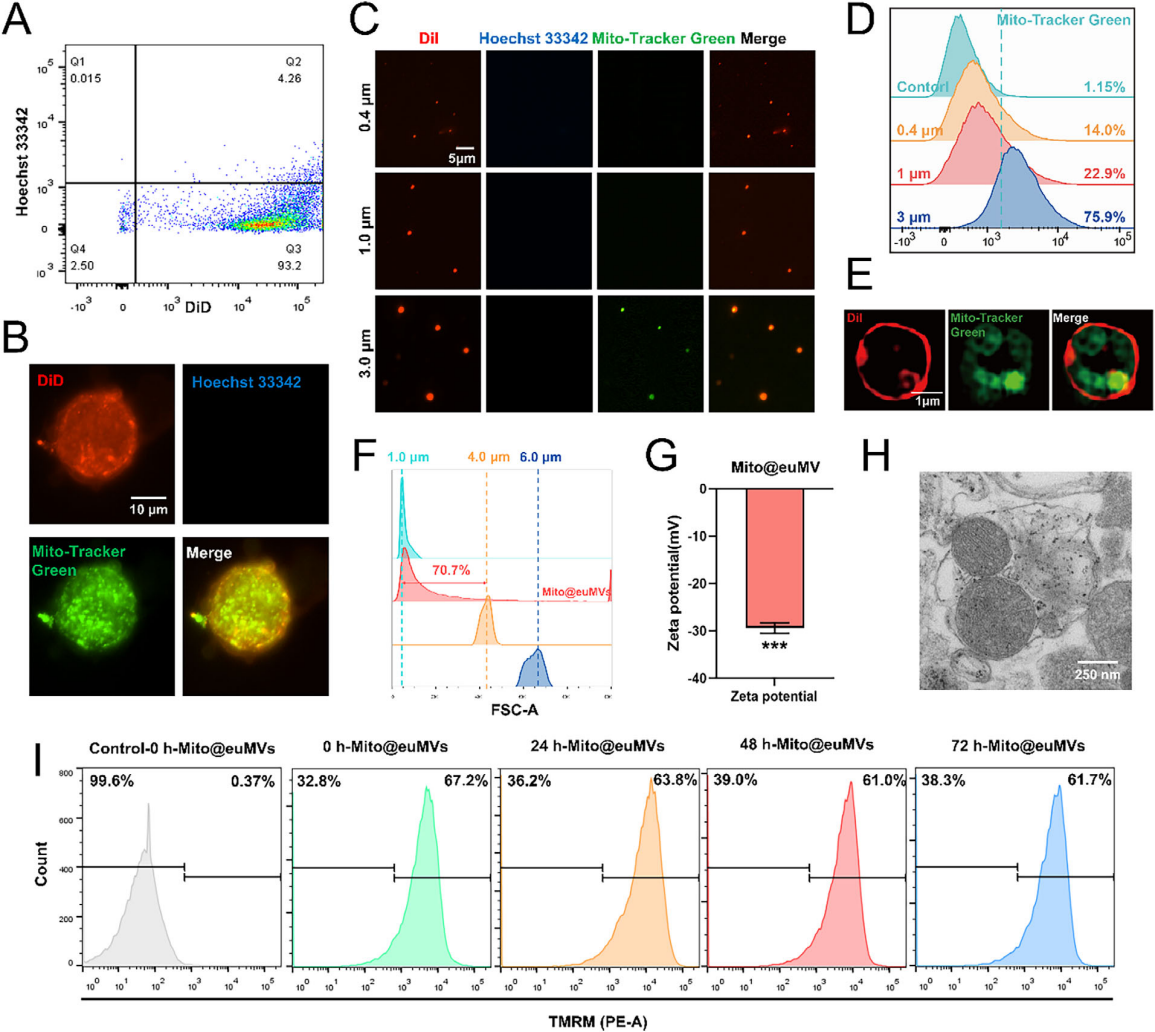
Mito@euMVs contain active mitochondria. A) The enucleation rate of MSCs assessed by flow cytometry. Data were analyzed using Flowjo software. The cell membrane labeled with DiD, nuclei labeled with Hoechst 33342. B) Fluorescence images showing enucleated MSCs in suspension, with the cell membrane labeled with DiD, nuclei labeled with Hoechst 33342, and mitochondria within the cells labeled with Mito‐Tracker Green. C) Fluorescence images illustrating enucleated MSCs‐derived MVs (euMVs) of different diameters (0.4 µm, 1.0 µm, 3.0 µm) prepared via one‐step extrusion method. The euMV membrane was labeled with DiI (red), nuclei with Hoechst 33342 (blue), and mitochondria with Mito‐Tracker Green (green) to visualize the encapsulation of mitochondria in euMVs of varying diameters. D) The proportion of euMVs of different diameters (0.4 µm, 1.0 µm, 3.0 µm) containing mitochondria analyzed by flow cytometry. The mitochondria were labeled with Mito‐Tracker Green. E) Representative fluorescence images using a HIS‐SIM showing Mito@euMVs, with the membrane of Mito@euMVs labeled with DiI (red) and the mitochondria within Mito@euMVs stained with Mito‐Tracker Green (green). F) Size distribution of Mito@euMVs determined by flow cytometry. The FSC intensity of Mito@euMVs was compared with that of particle‐size calibration standards (1.0 µm, 4.0 µm, 6.0 µm). G) Zeta potential of Mito@euMVs measured by dynamic light scattering (DLS) technique using a Zetasizer Nano ZS analyzer and analyzed by Dispersion Technology Software. H) Transmission electron microscopy images of Mito@euMVs containing mitochondria. I) Mitochondrial activity in Mito@euMVs assessed by flow cytometry. Control‐0 h‐Mito@euMVs refers to Mito@euMVs that were not labeled with TMRM, whereas 0 h/24 h/48 h/72 h‐Mito@euMVs are Mito@euMVs labeled with TMRM and analyzed at indicated time points postextrusion. The data was analyzed in Flowjo. n = 3, ***p < 0.001. All data are presented as the mean ± SD.

The nuclei of MSCs were first removed by cytochalasin B and centrifugation to avoid the unpredictable risks of genetic material in MSCs’ nuclei in subsequent applications and to mimic the absence of nuclear components in natural extracellular vesicles. The flow cytometry results demonstrated that the MSC enucleation rate exceeded 90% (**Figure** **1**A). The obtained enucleated MSCs retained their intact plasma membrane. More importantly, they contained active mitochondria labeled with Mito‐Tracker Green (Figure 1B). Next, liposome extrusion technology was used to prepare euMVs derived from enucleated MSCs. In this study, PC membranes with pore sizes of 0.4 µm, 1.0 µm, and 3.0 µm were selected to prepare euMVs of different sizes because mitochondria typically range in size from 0.5 to 1.0 µm. As shown in Figure 1C, euMVs were successfully produced with all selected PC membranes. Notably, the 3.0‐µm euMVs contained the most active mitochondria. Flow cytometry analysis revealed that 75.9% of the 3.0‐µm euMVs contained mitochondria, compared to 22.9% for the 1.0‐µm euMVs and 14.0% for the 0.4‐µm euMVs (Figure 1D), which was consistent with the fluorescence imaging observations. Thus, 3.0‐µm euMVs, hereafter referred to as Mito@euMVs, were used in all subsequent experiments. Representative fluorescence images obtained using a High Intelligent and Sensitive Structured Illumination Microscope (HIS‐SIM) confirmed that Mito@euMVs retained an intact membrane structure and successfully encapsulated mitochondria, as indicated by Mito‐Tracker Green labeling (Figure 1E). The size distribution of Mito@euMVs was measured by flow cytometry using flow cytometric particle size‐calibrated microspheres. According to the results, 70.7% of the Mito@euMVs had a particle size between 1.0 and 4.0 µm (Figure 1F). Mito@euMVs had a Zeta potential of ‐29.43 mV, indicating that they were relatively stable and unlikely to aggregate (Figure 1G). The presence of mitochondria from MSCs within the Mito@euMVs was further confirmed by transmission electron microscopy (TEM) (Figure 1H). The activity of mitochondria in Mito@euMVs is essential for their potential therapeutic effects. Therefore, we examined changes in the mitochondrial membrane potential of Mito@euMVs using tetramethylrhodamine methyl ester (TMRM) fluorescent dye by flow cytometry. The fluorescence intensity of TMRM‐labeled Mito@euMVs confirmed that the mitochondria in Mito@euMVs remained active for at least 72 h (Figure 1I). Meanwhile, naked mitochondria isolated from MSCs were readily depolarized when left unprotected (Figure S2A, Supporting Information). Considering that Mito@euMVs may contain other active components, we employed label‐free quantitative proteomics technology to analyze the components of Mito@euMVs. It was found that Mito@euMVs and MSCs both contained some pro‐healing factors, such as vascular endothelial growth factor D (VEGFd) and tissue inhibitor of metalloproteinase 1 (Timp1) (Figure S1A, Supporting Information). Thus, besides delivering active mitochondria, Mito@euMVs may also promote wound repair through supplementing pro‐healing factors. Moreover, the proteomic analysis revealed that Mito@euMVs contained a large amount of mitochondria proteins (Figure S1A, Supporting Information). By classifying the total proteins of Mito@euMVs based on subcellular localization, 14% of the proteins were localized in mitochondria (Figure S1B, Supporting Information). Gene Ontology (GO) functional annotation analysis also indicated that Mito@euMVs are rich in proteins associated with mitochondrial structure and function (Figure S1C, Supporting Information). Moreover, GO functional annotation analysis revealed that Mito@euMVs share similar profiles with MSCs in modulating oxidative stress and immune responses (Figure S1D, Supporting Information). We also performed the Protein‐Protein Interaction (PPI) analysis of highly expressed proteins in Mito@euMVs and showed that Mito@euMVs are mainly associated with metabolism, the tricarboxylic acid cycle, reactive oxygen species metabolism (Figure S1E, Supporting Information). Together, these results indicated that we successfully prepared Mito@euMVs that encapsulated active mitochondria with intact structures through the enucleation and extrusion of MSCs and therefore have the potential to transfer mitochondria to target cells.

### 2.2 Mito@euMVs Inhibit and Rescue the Hyperglycemia‐Induced Senescent Phenotype of Fibroblasts and HUVECs

**Figure 2:**
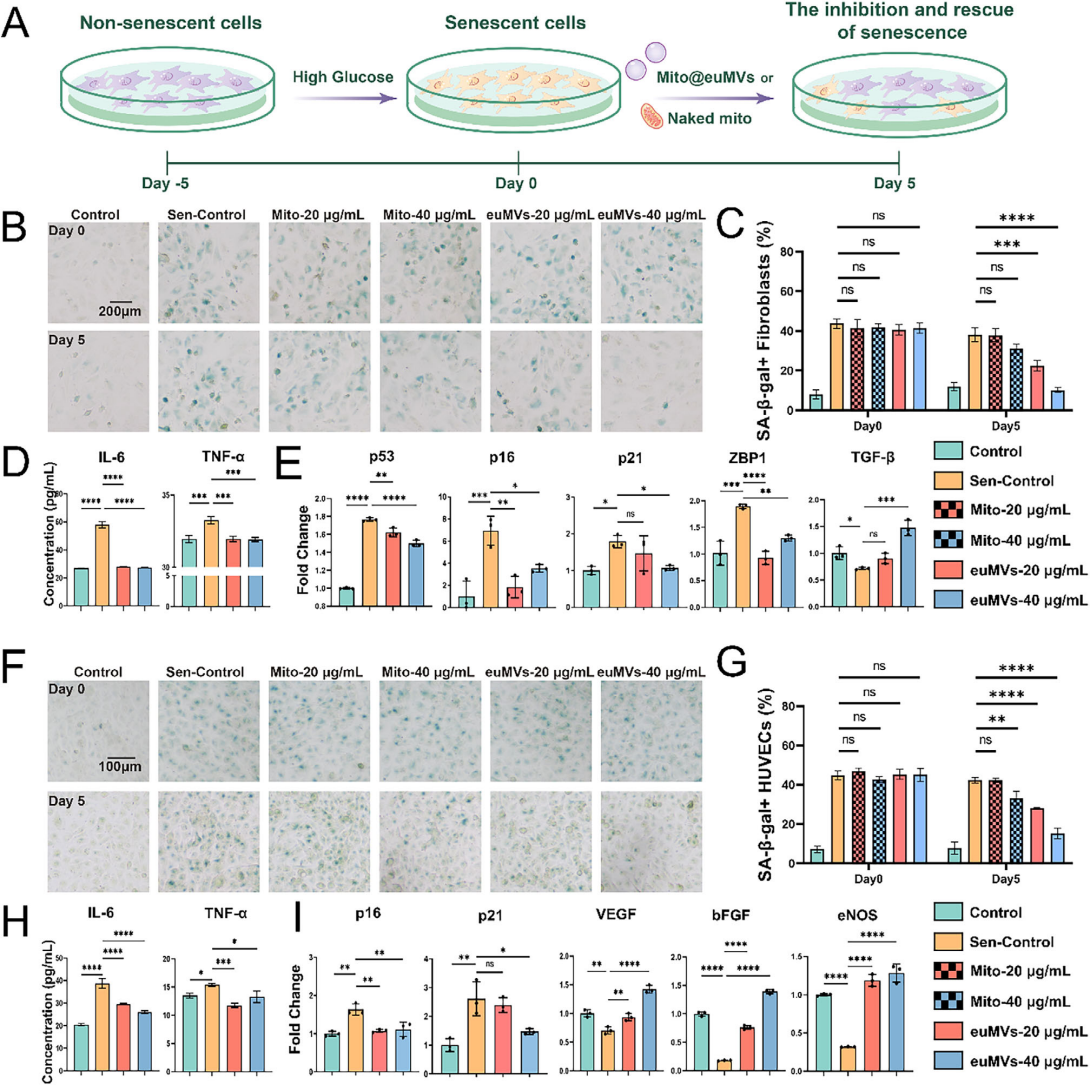
Mito@euMVs inhibit and rescue the senescent phenotypes of fibroblasts and HUVECs. A) The schematic diagram of the induction and treatment of senescent cells. Fibroblasts or HUVECs were induced to display a senescent phenotype by exposing them to high‐glucose DMEM with a glucose concentration of 35 mM for 5 days. Subsequently, senescent cells were treated with 20 µg mL^−1^ and 40 µg mL^−1^ Mito@euMVs or naked mitochondria isolated from MSCs for 5 days, respectively. B) Representative bright‐field images showing the SA‐β‐Gal expression in hyperglycemia induced senescent fibroblasts with/without receiving treatment of Mito@euMVs (euMVs‐20µg mL^−1^ and euMVs‐40µg mL^−1^) or naked mitochondria isolated from MSCs. C) The proportion of SA‐β‐Gal‐positive fibroblasts. D) Concentration of secreted key senescent associated secretory phenotype (SASP) factors IL‐6 and TNF‐α measured by ELISA in conditioned media from senescent fibroblasts treated with Mito@euMVs, nontreated senescent fibroblasts, and control fibroblasts. E) The expression of senescence related genes (*p53, p16*, *p21*, and *ZBP1*,) and pro‐repairing gene (*TGF‐β*) in fibroblasts analyzed by qPCR. F) Representative bright‐field images showing the SA‐β‐Gal expression in hyperglycemia induced senescent HUVECs with/without receiving treatment of Mito@euMVs (euMVs‐20 µg mL^−1^ and euMVs‐40 µg mL^−1^) or naked mitchondria isolated from mitochondria. G) The proportion of SA‐β‐Gal‐positive HUVECs. H) Concentration of secreted key SASP factors IL‐6 and TNF‐α measured by ELISA in conditioned media from senescent HUVECs treated with Mito@euMVs, nontreated senescent HUVECs, and control HUVECs. I) The expression of senescence related genes (*p16* and *p21*) and angiogenic genes (*VEGF, bFGF*, and *eNOS*) in HUVECs analyzed by qPCR. The Control group represents non‐senescent cells cultured at a glucose concentration of 5.5 mM, the Sen‐Control group represents senescent cells cultured at a glucose concentration of 35 mM, the Mito‐20 µg mL^−1^ and Mito‐40 µg mL^−1^ groups represent senescent cells subjected to treatment with 20 µg mL^−1^ and 40 µg mL^−1^ naked mitochondria, while the euMVs‐20 µg mL^−1^ and euMVs‐40 µg mL^−1^ groups represent senescent cells subjected to treatment with 20 µg mL^−1^ and 40 µg mL^−1^ Mito@euMVs, respectively. n = 3, *p<0.05, **p<0.01, ***p < 0.001, ****p<0.0001. All data are presented as the mean ± SD.

To study the regulatory effect of Mito@euMVs on the senescent phenotypes of key cells in diabetic wounds, we induced cellular senescence by culturing fibroblasts and HUVECs in culture medium supplemented with 35 mM glucose (**Figure** **2**A). The increase in the number of senescent‐associated beta‐galactosidase (SA‐β‐Gal)‐positive cells cultured under hyperglycemic condition compared to those cultured under normal condition confirmed that hyperglycemia was an effective inducer of cellular senescence (Figures S3A, B and S4A, B, Supporting Information). Both senescent fibroblasts and HUVECs were capable of uptaking DiI‐labeled Mito@euMVs in a dose‐dependent manner (Figures S3C, D and S4C, D, Supporting Information). The efficiency exceeded 90% when the concentration was higher than 20 µg mL^−1^, and surpassed 95% at 40 µg mL^−1^. Therefore, we selected concentrations of 20 and 40 µg mL^−1^ for further experiments. We next investigated the functional changes associated with senescence in fibroblasts and HUVECs after Mito@euMVs treatment. After treatment with Mito@euMVs for 5 days, the proportion of senescence‐associated β‐galactosidase (SA‐β‐Gal)‐positive cells in fibroblasts (Figure 2B,C) and HUVECs (Figure 2F,G) was significantly reduced. We also compared the effects of Mito@euMVs and naked mitochondria isolated from MSCs on the inhibition and rescue of cellular senescence. Our results showed that treatment with naked mitochondria did not reduce the proportion of SA‐β‐Gal‐positive fibroblasts (Figure 2B,C). While a high concentration of naked mitochondria (40 µg mL^−1^) reduced the proportion of SA‐β‐Gal‐positive HUVECs, the effect was less pronounced than that of Mito@euMVs (Figure 2F,G). These findings highlight the superior therapeutic efficiency of Mito@euMVs in mitigating cellular senescence compared to naked mitochondria. Since both senescent fibroblasts and HUVECs were capable of taking up naked mitochondria (Figure S2B,D, Supporting Information), the enhanced inhibition of cellular senescence is likely due to the protection provided by euMvs to the enclosed mitochondria. In addition to being SA‐β‐Gal‐positive, senescent cells are also characterized by high secretion of proinflammatory cytokines (e.g., IL‐6, IL‐8, IL‐1β, and TNF‐α) and matrix metalloproteinases (e.g., MMP‐1 and MMP‐10), which are collectively referred to as the senescent‐associated secretory phenotype (SASP).^[^ ^20^ ^]^ Therefore, changes in the secretion of two typical SASP factors, IL‐6 and TNF‐α, were detected by enzyme‐linked immunosorbent assay (ELISA). As expected, the levels of both cytokines were significantly decreased in senescent fibroblasts (Figure 2H) and HUVECs (Figure 2D) treated with Mito@euMVs. In addition, Mito@euMVs markedly downregulated the expression of senescence‐related genes, including *p53*,^[^ ^21^ ^]^ *p16, p21*, and *ZBP1* ^[^ ^22^ ^]^ in fibroblasts (Figure 2E). It is also noted that Mito@euMVs upregulated the expression of transforming growth factor‐β (*TGF‐β*) in fibroblasts, which is a critical regulator in promoting wound healing. Likewise, the expression of senescence‐related genes (*p16* and *p21*) was significantly downregulated, while genes related to HUVEC function (*VEGF, bFGF*, and *eNOS*) was significantly upregulated after treatment with Mito@euMVs, indicating that Mito@euMVs improved the function of HUVECs (Figure 2I). In addition, the regulatory effects of Mito@euMVs on other cellular behaviors, such as proliferation and migration, were also examined. Unsurprisingly, Mito@euMVs promoted the migration (Figure S3E,F, Supporting Information) and proliferation (Figure S3G, Supporting Information) of senescent fibroblasts. Additionally, the in vitro tube‐forming capacity of HUVECs was enhanced by Mito@euMVs. The total length of the tubes in the 40 µg mL^−1^ Mito@euMVs group was significantly greater than that in the Control‐Sen group, signifying a substantially improved angiogenic tendency in vitro (Figure S4E,F, Supporting Information). Mito@euMVs also promoted the chemotactic migration (Figure S4G,H, Supporting Information) and proliferation (Figure S4I, Supporting Information) of senescent HUVECs. It is noteworthy that non‐senescent fibroblasts and HUVECs cultured under normal conditions were capable of internalizing Mito@euMVs (Figure S5A, F, Supporting Information). Additionally, Mito@euMVs promoted the migration(Figure S5B, C, G, H, Supporting Information) and proliferation (Figure S5E, J, Supporting Information) of these cells. Thus, given that hyperglycemia did not induce senescence in all cells, the observed reduction in the senescent phenotype of fibroblasts and HUVECs following Mito@euMVs treatment may result from both the inhibition of non‐senescent cells transitioning to senescence and the rescue of existing senescent cells. These results collectively demonstrated that Mito@euMVs effectively inhibited and rescued the senescence phenotypes of fibroblasts and HUVECs and promoted their functions related to wound healing.

### 2.3 Mito@euMVs Inhibit and Rescue Hyperglycemia‐Induced Cellular Senescence Through Transferring Mitochondria

**Figure 3:**
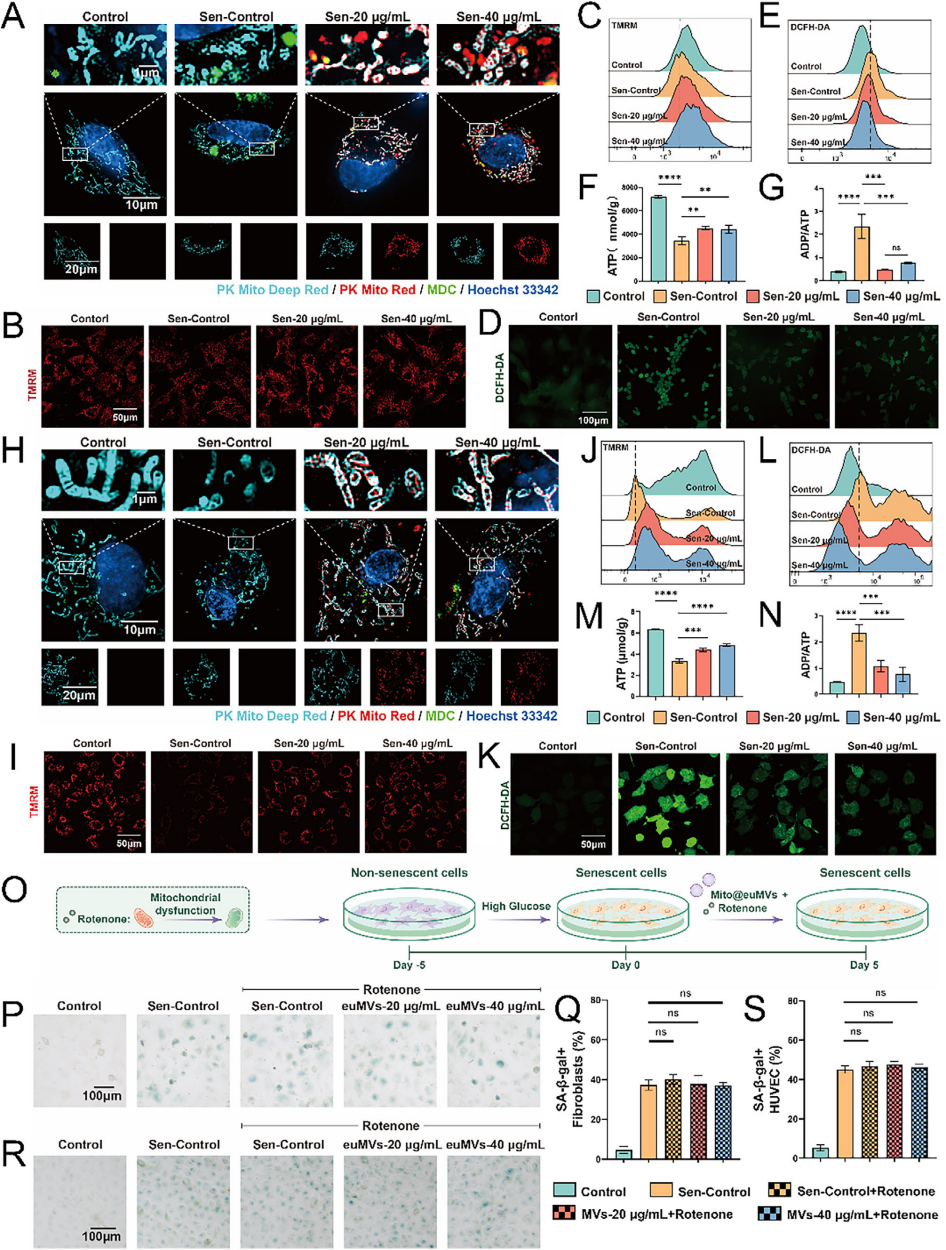
Mito@euMVs inhibit and rescue cellular senescence through transferring mitochondria. A) Representative fluorescence images using a HIS‐SIM showing the transfer of MSC mitochondria to fibroblasts by Mito@euMVs. The mitochondria of fibroblasts were fluorescently labeled with PK Mito Deep Red (cyan), the mitochondria within the Mito@euMVs were labeled with PK Mito Red (red), and the autophagosomes were stained with an Autophagy Staining Assay Kit with MDC (green), with nuclei counterstained with Hoechst 33342(blue). The fibroblasts were treated with PK Mito Red‐labeled Mito@euMVs for 24 h before being photographed. B) Representative fluorescence images showing the mitochondria membrane potential of fibroblasts determined via TMRM staining. C) The fluorescence intensity of TMRM in individual fibroblasts assessed using flow cytometry. D) Representative fluorescence images showing the intracellular ROS in fibroblasts detected by ROS probe DCFH‐DA. E) The fluorescence intensity of DCFH‐DA in individual fibroblasts assessed using flow cytometry. F) ATP production in fibroblasts measured by chemiluminescence. G) The ADP/ATP ratio in fibroblasts measured by chemiluminescence. H) Representative fluorescence images using a HIS‐SIM showing the transfer of MSC mitochondria to HUVECs by Mito@euMVs. The mitochondria of HUVECs were fluorescently labeled with PK Mito Deep Red (cyan), the mitochondria within the Mito@euMVs were labeled with PK Mito Red(red), and the autophagosomes were stained with an Autophagy Staining Assay Kit with MDC (green), with nuclei counterstained with Hoechst 33342(blue). The HUVECs were treated with PK Mito Red‐labeled Mito@euMVs for 24 h before being photographed. I) Representative fluorescence images showing the mitochondria membrane potential of HUVECs determined via TMRM staining. J) The fluorescence intensity of TMRM in individual HUVECs assessed using flow cytometry. K) Intracellular ROS levels in HUVECs detected by ROS probe DCFH‐DA. L) The fluorescence intensity of DCFH‐DA in individual HUVECs assessed using flow cytometry. M) ATP production in HUVECs measured by chemiluminescence. N) The ADP/ATP ratio in HUVECs measured by chemiluminescence. O)The schematic diagram of the induction and treatment of senescent cells using Mito@euMVs in the presence of rotenone. Fibroblasts or HUVECs were induced to display a senescent phenotype by exposing them to high‐glucose DMEM with a glucose concentration of 35 mM for 5 days. Subsequently, senescent cells were treated with/without Mito@euMVs in the presence of rotenone for 5 days. P) Representative bright‐field images showing the SA‐β‐Gal expression in hyperglycemia induced senescent fibroblasts with/without receiving treatment of Mito@euMVs in the presence of rotenone. Q) The proportion of SA‐β‐Gal‐positive fibroblasts. R) Representative bright‐field images showing the SA‐β‐Gal expression in hyperglycemia induced senescent HUVECs with/without receiving treatment of Mito@euMVs in the presence of rotenone. S) The proportion of SA‐β‐Gal‐positive HUVECs. The Control group represents non‐senescent cells cultured at a glucose concentration of 5.5 mM, the Sen‐Control group represents senescent cells cultured at a glucose concentration of 35 mM, the rotenone groups represent senescent cells cultured with 1.5 µM rotenone, while the Sen‐20 µg mL^−1^ and Sen‐40 µg mL^−1^ groups represent senescent cells receiving 20 µg mL^−1^ and 40 µg mL^−1^ Mito@euMVs treatment, respectively. n = 3, **p<0.001, ***p < 0.001, ****p<0.0001. All data are presented as the mean ± SD.

We next asked whether the rescued cellular senescence was associated with the mitochondria in the Mito@euMVs. To address this question, we investigated the transfer of MSC mitochondria from Mito@euMVs into recipient cells. Mitochondria in Mito@euMVs were labeled with PK Mito Red(red), while mitochondria of the recipient fibroblasts and HUVECs were labeled with PK Mito Deep Red (cyan). Senescent fibroblasts and HUVECs treated with Mito@euMVs for 24 h were visualized using a HIS‐SIM. Fluorescence images revealed that Mito@euMVs’ enclosed mitochondria (red) were transferred into recipient fibroblasts (**Figure** **3**A) and HUVECs (Figure 3H). Furthermore, the partially overlapping red and cyan fluorescence indicated that the mitochondria from Mito@euMVs integrated into the recipient cells’ mitochondrial network. Notably, a small fraction of mitochndrial from Mito@euMVs was colocalized with autophagosomes (MDC, green). These results suggested that mitochondria transferred to fibroblasts and HUVECs by Mito@euMVs retained their activity and were expected to function continuously within the cells.

Senescent cells typically exhibit impaired mitochondrial function, characterized by decreased mitochondrial membrane potential,^[^ ^5a^ ^]^ excessive reactive oxygen species (ROS) production, and impaired ATP production capacity. Thus, we further examined the impact of Mito@euMVs on the mitochondrial function of senescent fibroblasts and HUVECs. The fluorescence intensity of TMRM in individual cells revealed that the mitochondrial membrane potential significantly decreased in senescent cells compared to that in cells (Control). After Mito@euMVs treatment, there was a substantial increase in the mitochondrial membrane potential, reaching a level almost comparable to that observed in non‐senescent fibroblasts (Figure 3B,C) and HUVECs (Figure 3I,J). Additionally, Mito@euMVs mitigated the abnormally increased ROS production in senescent fibroblasts (Figure 3D,E) and HUVECs (Figure 3K,L). We also compared the effects of Mito@euMVs and naked mitochondria on regulating TMRM and ROS production in senescent fibroblasts and HUVECs. Our results showed that while naked mitochondria effectively increased TMRM levels and decreased ROS in senescent fibroblasts and HUVECs, the effect was less pronounced than that observed with Mito@euMVs (Figure S2C, E, F‐I, Supporting Information). Given that mitochondria are pivotal for cellular energy production, the levels of intracellular ATP and its decomposition product ADP serve as crucial indicators of mitochondrial function. Thus, changes in ATP production and ADP/ATP ratio were detected using a chemiluminescence method. The results showed that ATP production was markedly reduced in both senescent fibroblasts and HUVECs compared to non‐senescent cells, while treatment with Mito@euMVs led to an increase in ATP production (Figure 3F,M). Moreover, a notable increase in the ADP/ATP ratio was observed in senescent cells, which decreased significantly following Mito@euMVs treatment (Figure 3G,N). As ATP synthesis and decomposition are dynamically balanced in healthy cells, Mito@euMVs restored ATP synthesis in the cell, resulting in a decrease in the ADP/ATP ratio. Collectively, these findings confirmed that Mito@euMVs successfully transferred active mitochondria into recipient cells, significantly improving the mitochondrial functions of senescent fibroblasts and HUVECs. To further investigate the role of mitochondria in rescuing cellular senescence by Mito@euMVs, we evaluated cellular senescence under hyperglycemic conditions after inhibiting mitochondrial function with rotenone (Figure 3O). Notably, rotenone significantly reversed the effects of Mito@euMVs, as evidenced by a similar percentage of SA‐β‐Gal‐positive cells in the rotenone‐treated groups compared to the Sen‐Control group (Figure 3P–S). This finding indicated that the inhibition and rescue of cellular senescence by Mito@euMVs is primarily attributed to the mitochondria enclosed within the euMVs.

### 2.4 Dissolving PVA Microneedle Patches Facilitate the Transdermal Delivery of Mito@euMVs

**Figure 4:**
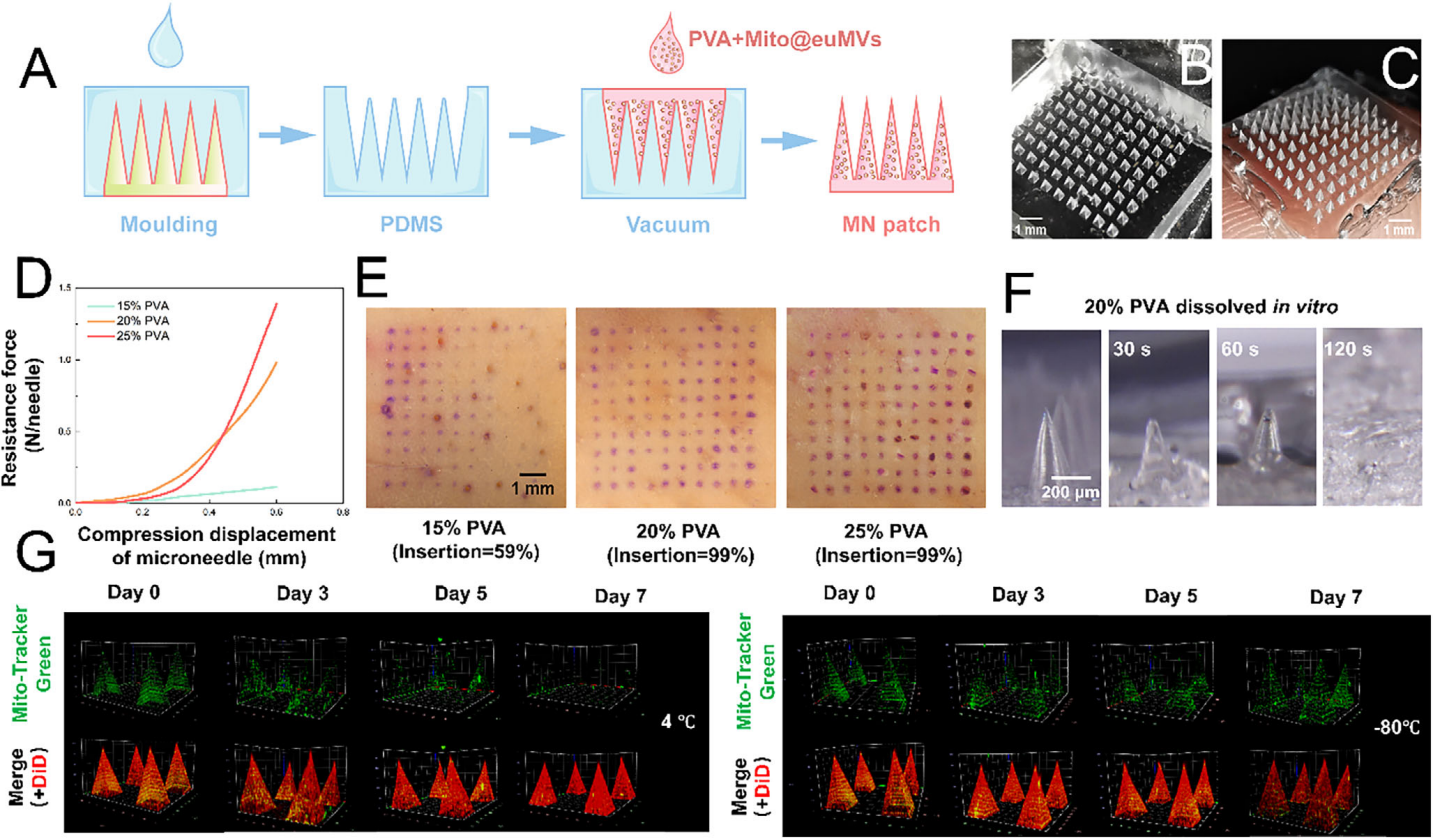
Preparation and characterization of dissolving PVA microneedle patches. A) Schematic of workflow depicting the preparation of dissolving PVA microneedle (MN) patch loaded with Mito@euMVs. B) Representative optical image of PDMS mold for microneedle patch. C) Representative optical image of PVA‐MN patch. D) Mechanical behavior of 15%, 20%, 25% PVA‐MN patches against compressive displacement. E) Penetration of porcine ear skin by 15%, 20%, 25% PVA‐MN patches. The penetrated skins were stained by crystal violet. F) Representative optical microscope images showing the rapid dissolution of 20% PVA‐MNs upon insertion into 15% GelMA hydrogel within 120 s. G) Representative fluorescence images demonstrating the stability of Mito@euMVs and the overall structural integrity of mitochondria within Mito@euMVs loaded in PVA microneedles at 4°C and ‐80°C. The membrane of Mito@euMVs was labeled by DiD and mitochondria were labeled by Mito‐Tracker Green. n = 3.

The efficient delivery of Mito@euMVs to wounded skin is crucial for ensuring their therapeutic efficacy. Recently, microneedles (MNs) have gained considerable attention as transdermal delivery tools owing to their ability to precisely and controllably deliver drugs to specific skin layers, as well as their advantages, such as minimal invasiveness, high efficiency, and cost‐effectiveness.^[^ ^23^ ^]^ In this study, we opted for a dissolving polyvinyl alcohol (PVA)‐MN patch as a carrier for the rapid and efficient delivery of Mito@euMVs. The dissolving PVA‐MN patch loaded with Mito@euMVs was prepared according to the process shown in **Figure** **4**A. The corresponding PDMS mold is shown in Figure 4B. Each tip is designed as a right square pyramid, with a height of 600 µm and a bottom square side length of 300 µm. Each patch comprised a total of 10×10 needle tips. The demolded PVA‐MN patch was in good accordance with the design (Figure 4C). It is necessary that the mechanical strength of the obtained PVA‐MN is sufficient to penetrate the skin. Thus, we performed a compression experiment (Figure 4D), which showed that 20% and 25% PVA‐MN had much better mechanical strength than 15% PVA‐MNs. The skin‐penetrating capability of MN was validated using porcine skin. As shown in Figure 4E, the tips of the 20% and 25% PVA‐MN demonstrated almost 100% penetration through porcine skin. Since 25% PVA‐MNs did not exhibit significantly better mechanical properties than 20% PVA‐MN, a 20% PVA‐MN patch was chosen for subsequent experiments. To assess the dissolution rate of the MNs, the alterations in the shape of the needle tips following their insertion into 15% gelatin methacrylate (GelMA) in vitro were captured with a stereomicroscope at different time points. The 20% PVA‐MN dissolved rapidly within 120 s, which ensured the efficient delivery of the Mito@euMVs (Figure 4F). We then examined the loading and distribution of Mito@euMVs in the MN. A large amount of DiD‐labeled Mito@euMVs were well distributed in each tip of the MN patch (Figure 4G). Additionally, given that MitoTracker‐Green covalently interacts with thiols on mitochondria proteins, the loss of its fluorescence indicates severely compromised structure integrity. Therefore, the presence of MitoTracker Green suggested that the mitochondria within the Mito@euMVs retained their overall structural integrity for at least 3 days at 4°C and 7 days at ‐80°C (Figure 4G). Undoubtedly, dissolving PVA‐MN patches may facilitate the transdermal delivery of Mito@euMVs in vivo.

### 2.5 Mito@euMVs Promote the Healing of Diabetic Wounds

**Figure 5:**
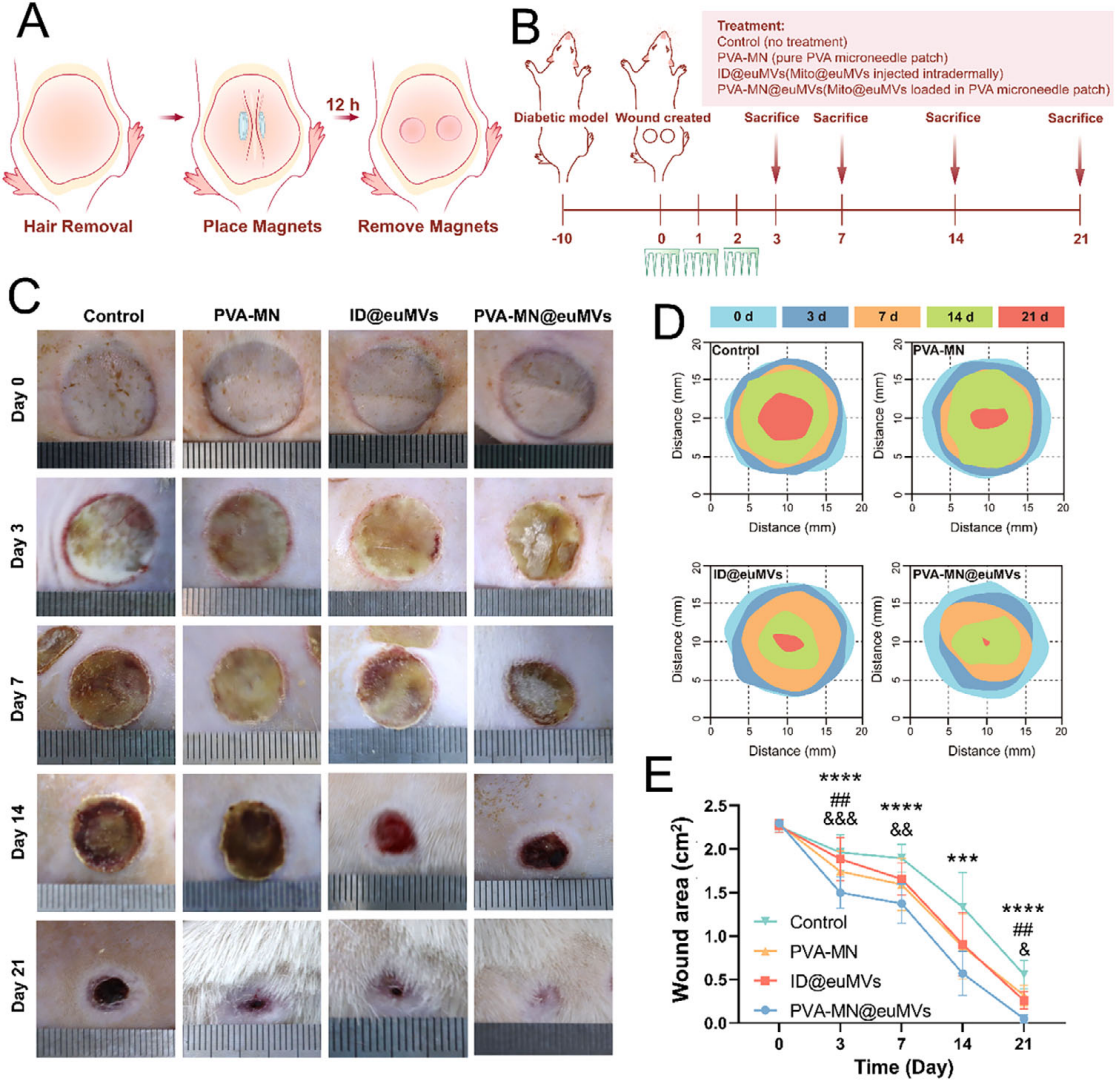
Mito@euMVs facilitate the healing of diabetic pressure sore rat. A) Schematic diagram of the procedure for inducing pressure sore wounds in diabetic rats. B) Diagram outlining the creation of the wound and subsequent steps involved in treating and sample harvest. C) Representative images showing the progression of healing in diabetic pressure sores among rats subjected to various treatments (n = 6). D) Comparative analysis of wound areas treated with various interventions on days 3, 7, 14, and 21. E) Assessment of wound areas on days 3, 7, 14, and 21 post treatments. n = 6, ***p < 0.001, ****p<0.0001 for PVA‐MN@euMVs vs Control, ##p<0.01 for ID@euMVs vs Control, &p<0.05, &&p<0.01, &&&p < 0.001 for PVA‐MN vs Control. All data are presented as the mean ± SD.

Next, we evaluated the therapeutic effects of Mito@euMVs by using a diabetic rat model with pressure sores (**Figure** **5**A, B). Wound assessments were conducted at 0,7,14, and 21 days posttreatment, after which changes in the wound area were calculated. Notably, among all the experimental groups, the group of Mito@euMVs‐loaded PVA‐MNs (PVA‐MN@euMVs) exhibited the most pronounced and rapid reduction in wound area (Figure 5C–E). After 21 days of treatment, the wounds in the PVA‐MN@euMVs group were completely closed. It is worth of mentioning that no significant difference in the healing rate between the PVA‐MN group and the Control group was observed, suggesting that the primary factor contributing to this outcome was the released Mito@euMVs rather than the MN patch itself. Additionally, the intradermal injection of Mito@euMVs (ID@euMVs) had less favorable effects on wound repair compared to the PVA‐MN@euMVs, highlighting the role of PVA‐MN patches in facilitating the precise delivery of Mito@euMVs to wounded tissue and thereby promoting efficient wound healing.

**Figure 6:**
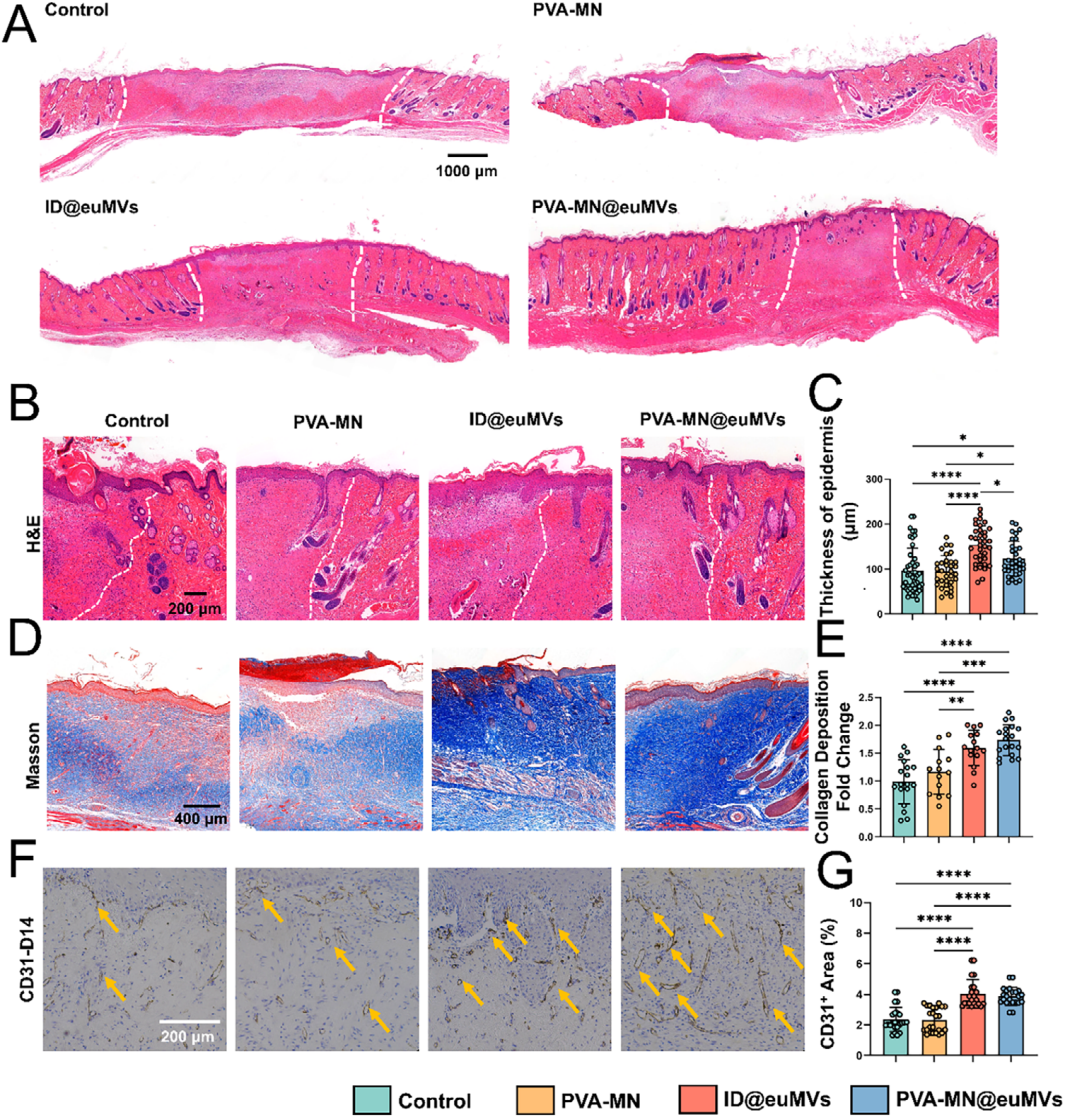
Histological assessment of the diabetic pressure sores in rats. A) Representative images of H&E staining showing the healing diabetic pressure sores on day 21. The wounded areas are demarcated by the white dotted lines. B) Representative images of H&E staining illustrating the wound edges on day 21. The left side of the white line indicates the wound area, while the right side represents intact skin. C) Analysis of the thickness of the newly formed epidermis in each group on day 21. D) Representative images of Masson staining of wounds on day 21. E) Analysis of relative collagen deposition in each group. F) Representative immunohistochemical staining for CD31 in wounds on day 14 revealed the presence of newly formed vessels, highlighted by yellow arrows. G) Analysis of CD31‐ positive areas in wounds in each group. n = 6, *p < 0.05, **p < 0.01, ***p < 0.001, ****p < 0.0001. All data are presented as the mean ± SD.

Similarly, H&E staining revealed that the PVA‐MN@euMVs group exhibited the most complete regeneration of skin appendages, including hair follicles and sweat glands (**Figure** **6**A). Furthermore, we measured the epidermal thickness of regenerative tissue in each group. As shown in Figure 6B,C, the thickness of the newly formed epidermis in the ID@euMVs and PVA‐MN@euMVs groups was significantly greater than that in the other two groups. Additionally, the proportion of inflammatory cells infiltrating the wound site was lower in the ID@euMVs and PVA‐MN@euMVs groups (Figure S6, Supporting Information). To observe the deposition of collagen in the wound, Masson trichrome staining was applied to the skin tissue samples on day 21. The results indicated that although new collagen deposition and regenerated tissue were present in all groups, the collagen deposition in the ID@euMVs and PVA‐MN@euMVs groups was denser (Figure 6D,E). We also assessed angiogenesis during the healing process by using the neovascularization marker CD31. The immunohistochemical (IHC) results for CD31 demonstrated that the ID@euMVs and PVA‐MN@euMVs induced the most robust neovascularization, with the densest blood vessels among all four groups (Figure 6F,G).

Taken together, these results demonstrated that ID@euMVs and PVA‐MN@euMVs, especially PVA‐MN@euMVs, expedited the repair of diabetic chronic pressure sore wounds through promoting wound closure and tissue regeneration.

### 2.6 Mito@euMVs Rescue Cellular Senescence at the Wound Site

**Figure 7:**
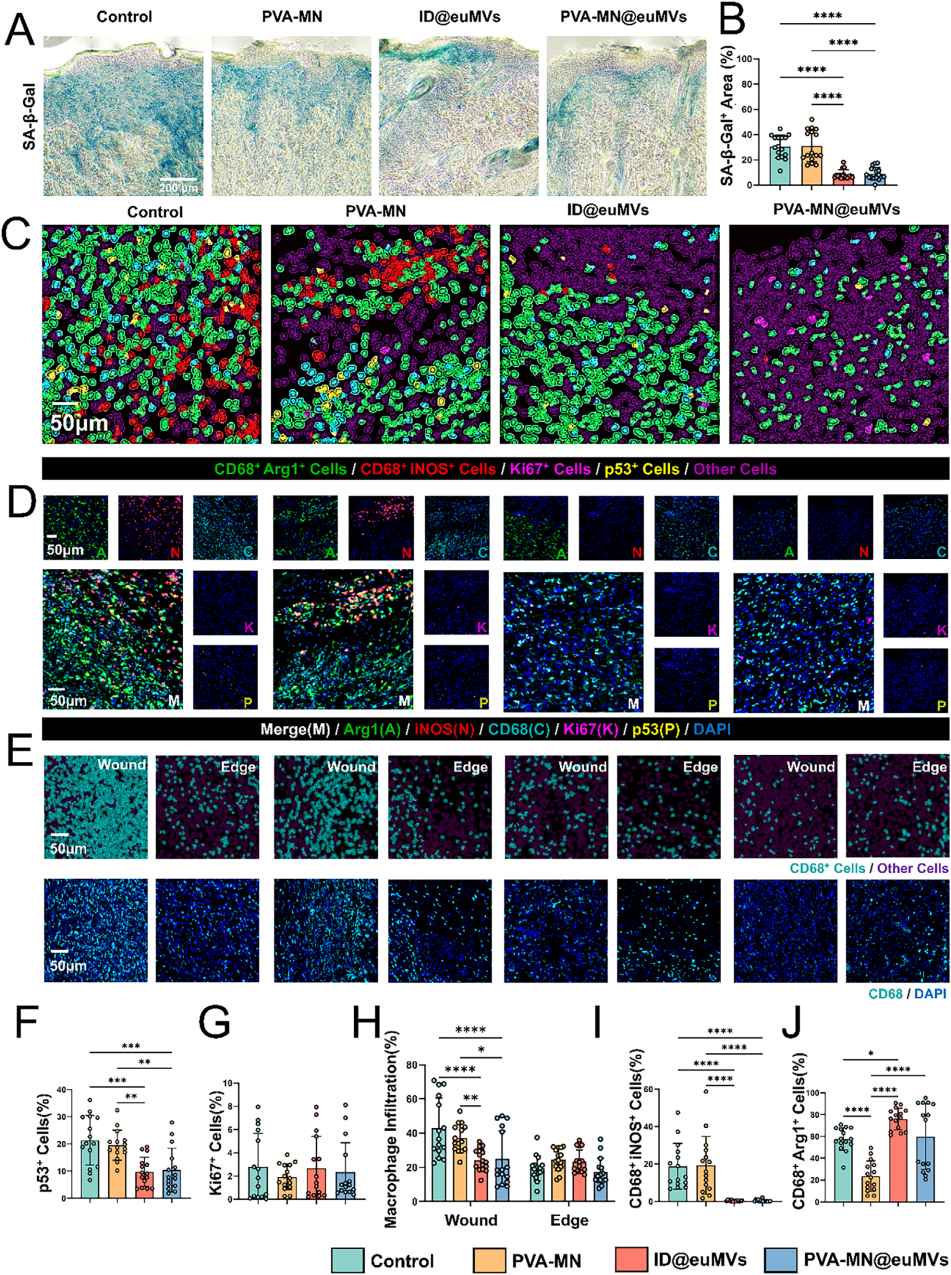
Assessment of cellular senescence at wounded sites in diabetic pressure sores receiving various treatment. A‐B) Representative staining images showing SA‐β‐Gal (a) and the corresponding quantification of SA‐β‐Gal positive area proportion per unit area (b) on day 21. C) Representative images of different cell phenotypes in wounded tissue. Different cells were segmented and assigned with distinct colors via the QuPath software. D) Representative images of multiplex immunofluorescence images showing Arg1, iNOS, CD68, Ki67, and p53 staining. E) Representative images of macrophage infiltration in wound and edge areas in each group. Macrophages were identified by their CD68 positivity. F) Quantitative analysis of p53^+^ cells in wounds in each group. G) Quantitative analysis of Ki67^+^ cells in wounds in each group. H) Quantitative analysis of macrophage infiltration in wound and edge areas in each group. I) Quantitative analysis of CD68^+^iNOS^+^ cells in wounds in each group. J) Quantitative analysis of CD68^+^Arg1^+^ cells in wounds in each group. n = 6, *p < 0.05, **p < 0.01, ***p < 0.001, ****p < 0.0001. All data are presented as the mean ± SD.

In the aforementioned in vitro experiments, we validated the capability of Mito@euMVs to inhibit and rescue the hyperglycemia‐induced senescent phenotype of fibroblasts and HUVECs. To further substantiate the impact of Mito@euMVs on tissue in vivo, we examined the levels of SA‐β‐Gal, the well‐established marker of cell senescence, in tissue sections at wound sites. As illustrated in **Figure** **7**A,B, wounds treated with either PVA‐MN@euMVs or ID@euMVs exhibited a lower proportion of SA‐β‐Gal‐positive area, visualized as blue in the field, than those receiving either PVA‐MN or no treatment. This finding suggested that Mito@euMVs effectively inhibited the senescent state of diabetic wound tissue. To further explore the effects of Mito@euMVs on wounds, multiplex immunofluorescence (mIHC) staining was used to simultaneously label multiple markers related to senescence. As shown in Figure 7C, different cells at the wound sites were segmented and assigned distinct colors via QuPath software. We counted the change in the proportion of p53^+^ cells, that is, the cells marked in yellow. Compared with those in the PVA‐MN and Control groups, the proportion of p53^+^ cells decreased significantly in the groups receiving ID@euMVs and PVA‐MN@euMVs treatments (Figure 7F). For the cell proliferation marker Ki67 (magenta), few Ki67^+^ cells were detected, and there was no significant difference in the proportion of Ki67^+^ cells among the groups (Figure 7G). In fact, as an early response to injury, resident dermal fibroblasts in the neighborhood of the wound proliferate during the initial 2–3 days. Afterward, they switch from the proliferative phenotype to the migratory phenotype, crawling into the wound bed and laying down a collagen‐rich matrix.^[^ ^24^ ^]^ Thus, it is reasonable that only a small number of fibroblasts were proliferating on day 7. In contrast, a significant increase in the proportion of Ki67^+^ cells was observed in the ID@euMVs and PVA‐MN@euMVs groups on day 3 (Figure S7A, B, Supporting Information). Considering that Mito@euMVs acted on both non‐senescent and senescent cells in vitro, the observed reduction in the senescent state of diabetic wound tissue following Mito@euMVs treatment may be attributed to both the prevention of non‐senescent cells transitioning to senescence and the rejuvenation of already‐ senescent cells. Recently, it has been reported that the presence of Arg1^+^ macrophages were suppressed in aged regenerating tissue, suggesting that these macrophages play important roles in age‐related immune response disparities during repair and regeneration.^[^ ^25^ ^]^ Thus, the macrophage subsets were analyzed by labeling them with the universal macrophage marker CD68 (cyan), the M1 macrophage marker iNOS (red), and the M2 macrophage marker Arg1 (green) (Figure 7D). Unsurprisingly, the proportion of CD68^+^iNOS^+^cells in both ID@euMVs and PVA‐MN@euMVs groups was markedly decreased compared to both the untreated group and the PVA‐MN group (Figure 7C,I). Meanwhile, the proportion of CD68^+^Arg1^+^ cells in wounds treated with ID@euMVs was significantly increased (Figure 7C,J). These results confirmed that Mito@euMVs effectively inhibited and rescued the senescent state of diabetic wound tissue. In addition, macrophages play an important role in modulating the local immune microenvironment and inflammation. Consequently, the reduction in macrophage (CD68^+^ cell) infiltration observed in the wounded area treated with ID@euMVs and PVA‐MN@euMVs (Figure 7E,H), along with the decreased presence of M1 (CD68^+^iNOS^+^) and increased presence of M2 (CD 68^+^Arg1^+^), indicated that Mito@euMVs contribute to resolving inflammation at wound sites, thereby offering additional benefits to the healing process.

### 2.7 Mito@euMVs Improve Mitochondrial Function in Wound Tissue

**Figure 8:**
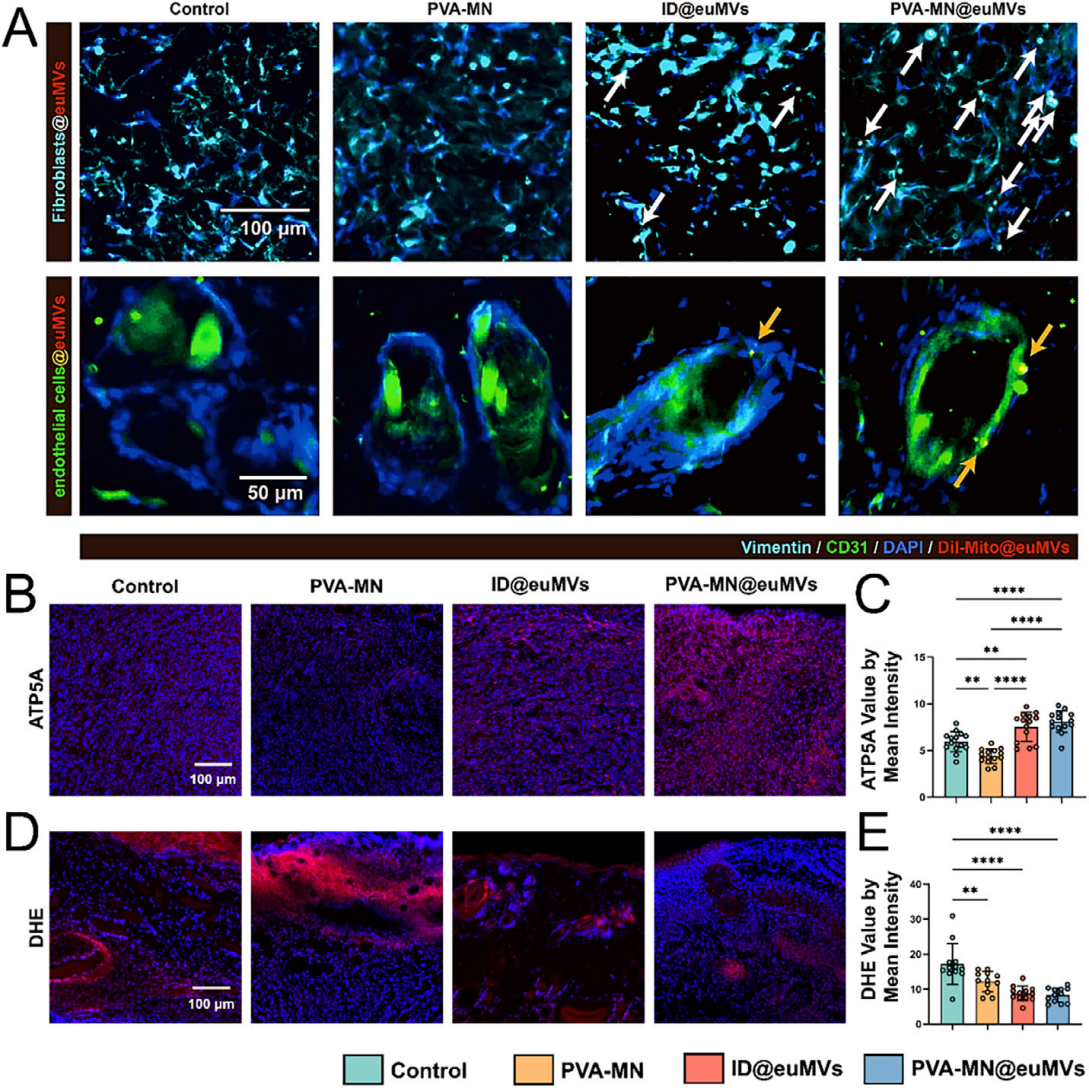
Mito@euMVs promote mitochondrial function in diabetic pressure sores in rats. A) Representative immunofluorescence images showing the cellular uptake of Mito@euMVs by fibroblasts and endothelial cells in vivo on day 1. Mito@euMVs were labeled with DiI (red), fibroblasts were stained with vimentin (cyan), and endothelial cells were marked with CD31 (green). Uptake of Mito@ MVs by fibroblasts is indicated by white arrows, while uptake of Mito@MVs by endothelial cells is indicated by orange arrows. B‐C) Representative immunofluorescence images showing ATP5A staining B) and the quantification of mean fluorescence intensity per unit area (C) on day 7. D‐E) Representative staining images for DHE (D) and the quantification of mean fluorescence intensity per unit area (E) on day 7. n = 6, *p<0.05, **p < 0.01, ***p < 0.001, ****p < 0.0001. All data are presented as the mean ± SD.

Next, we investigated the effects of Mito@euMVs on mitochondrial functions in wound tissue. To evaluate the cellular uptake of Mito@euMVs in vivo, we labeled Mito@euMVs with DiI (red) before administrating them to the wound sites via either ID or PVA‐MN. Tissue samples were harvested on day 1 and subjected to immunofluorescence staining using vimentin (cyan) as a fibroblast marker and CD31 (green) as a marker for HUVECs. Co‐localization of green and red fluorescence observed in both the ID@euMVs and PVA‐MN@euMVs groups confirmed the uptake of Mito@euMVs by firoblasts (**Figure** **8**A line 1, indicated by white arrow). Similarly, co‐localization of green and magenta fluorescence demonstrated their uptake by HUVECs (Figure 8A line 2, indicated by orange arrow). These result indicated that both fibroblasts and HUVECs were capable of internalizing Mito@euMVs in vivo. ATP5A is a catalytic subunit of the mitochondrial ATP synthase complex responsible for the production of ATP. Its expression serves as an indirect reflection of mitochondrial function in tissues. As expected, the expression of ATP5A in ID@euMVs and PVA‐MN@euMVs groups was significantly increased, indicating an improvement in mitochondrial function in wounds treated with Mito@euMVs (Figure 8B,C). One hallmark of diabetic wound microenvironments is abnormally high level of ROS, which is closely linked to mitochondrial dysfunction.^[^ ^26^ ^]^ Therefore, the level of ROS was also detected by using the ROS fluorescent probe dihydroethidium (DHE). The results showed notably lower ROS in the PVA‐MN@euMVs and ID@euMVs groups, signifying the capability ability of the Mito@euMVs to reduce ROS at the wound site. Interestingly, PVA‐MN reduced the level of ROS within the wound as well, albeit to a lesser extent than that observed in the PVA‐MN@euMVs and ID@euMVs groups (Figure 8D,E). Considering the link between ROS and tissue inflammation, this reduction suggests that Mito@euMVs alleviate the inflammatory status of wound tissue, which benefits the healing process. These findings collectively confirmed that ID@euMVs and PVA‐MN@euMVs possessed the capacity to improve mitochondrial function and activity in diabetic wounds, which is attributed to the transfer of mitochondria with Mito@euMVs, which may play a critical role in this rescue mechanism.

### 2.8 Mito@euMVs Alter the Proteome of Diabetic Wounds

**Figure 9:**
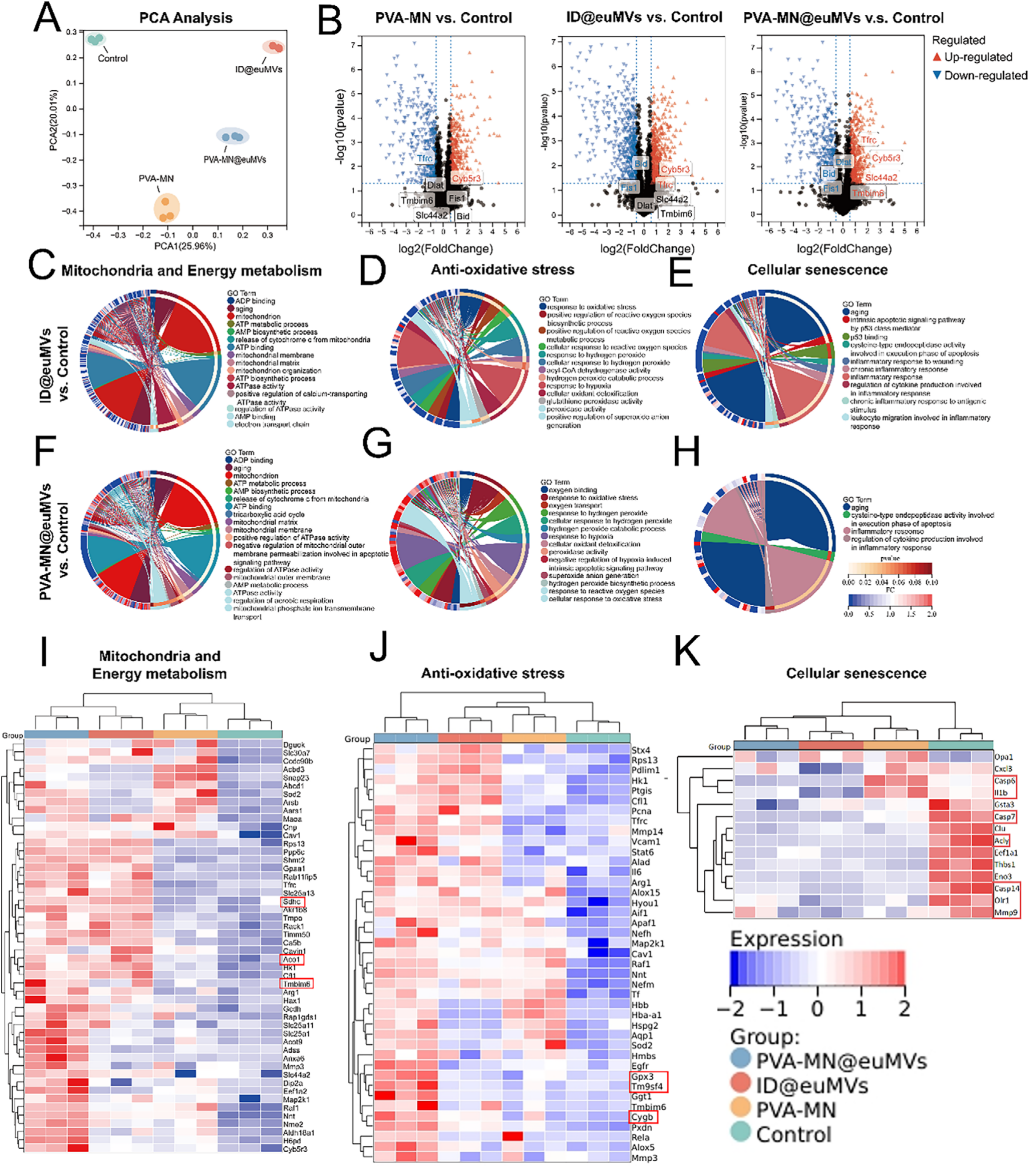
Proteomic profiling of wounded tissues in diabetic pressure sores receiving various treatments. A) The first (PC1) and second (PC2) principal components analysis (PCA) of the protein expression levels show a complete separation among the 4 groups. B) Volcano plots illustrating differentially expressed proteins (DEPs) where gray signifies nonsignificant genes, red indicates upregulated genes, and blue represents downregulated genes. C‐E) GO chord diagram displaying the relationship between DEPs and enriched pathway of mitochondria and energy metabolism (C), anti‐oxidative stress (D), and cellular senescence (E) between ID@euMVs and Control. F‐H) GO chord diagram displaying the relationship between DEPs and enriched pathway of mitochondria and energy metabolism (F), anti‐oxidative stress (G), and cellular senescence (H) between PVA‐MN@euMVs and Control. I‐K) Heatmap representation showcasing the DEPs involved in mitochondria and energy metabolism (I), anti‐oxidative stress (J), and cellular senescence (K). n = 3.

To elucidate the intricacies of how Mito@euMVs contribute to the healing of pressure sore wounds in diabetic rats, we employed label‐free quantitative proteomics technology to analyze wound tissue after receiving various treatments (PVA‐MN@euMVs, ID@euMVs, PVA‐MN, and no treatment). Principal component analysis (PCA) revealed that each group had a distinct protein expression profile, and the samples in each group could be clustered (**Figure** **9**A). Globally, a total of 3294, 3526, 3345, and 3402 proteins were identified in the PVA‐MN@euMVs, ID@euMVs, PVA‐MN, and Control groups, respectively. The Venn diagram of differentially expressed proteins (DEPs) revealed that 207 DEPs were shared in all paired comparisons, and 434, 243, and 241 DEPs were uniquely shown in ID@euMVs versus Control, PVA‐MN@euMVs versus Control, and PVA‐MN versus Control, respectively (Figure S8A, Supporting Information). The distribution of DEPs, highlighting their substantial variations compared to those in the Control group, is depicted in volcano plots (Figure 9B). Notably, in wounds treated with PVA‐MN@euMVs, proteins related to mitochondrial ATP production (e.g., Cyb5r3 and Slc44a2) and proteins related to mitochondrial fusion (e.g., Tfrc and Tmbim6) were significantly upregulated. Conversely, proteins associated with mitochondrial fission (Fis1) and apoptosis (Dlat, Bid) were significantly downregulated. These DEPs indicated an improvement in the mitochondrial functions of cells in the wounds. To gain more insight into the profile differences, we performed Kyoto Encyclopedia of Genes and Genomes (KEGG) pathway analysis. KEGG pathway analysis revealed that metabolic pathway, glucagon signaling pathway, cellular senescence, p53 signaling pathway, apoptosis, PI3K‐Akt signaling pathway, and PPAR signaling pathway were among the most affected pathways associated with upregulated DEPs in the PVA‐MN@euMVs and ID@euMVs groups (Figure S8B, Supporting Information). Considering that these pathways are closely related to energy metabolism, senescence, and inflammation, we subsequently conducted Gene Ontology (GO) enrichment analysis of the DEPs, focusing on GO terms related to mitochondria and energy metabolism (Figure 9C,F), antioxidative stress (Figure 9D,G), and cellular senescence (Figure 9E,H) in the ID@euMVs and PVA‐MN@euMVs groups compared to the control group. As shown in the GO chord diagram, the number of upregulated DEPs associated with mitochondria, energy metabolism, and antioxidative stress was significantly greater in the PVA‐MN@euMVs group than in the ID@euMVs group, with more pronounced differences. In contrast, DEPs in GO terms related to cellular senescence tended to be downregulated in both ID@euMVs and PVA‐MN@euMVs groups. A heatmap visualizing the expression levels of representative DEPs in the PVA‐MN@euMVs group provided more detailed information (Figure 9I–K). Typically, wounds treated with PVA‐MN@euMVs, exhibited significant upregulation of enzymes involved in the tricarboxylic acid cycle (e.g., Aco1 and Sdhc), the mitochondrial protective protein Tmbim6, and proteins that protect against oxidative damage (e.g., Gpx3, Cygb, Tm9sf4). Conversely, proteins associated with the SASP or apoptosis (e.g., Casp6/7/14, Mmp9, I11b, Olrl, and ACLY) were significantly downregulated in the PVA‐MN@euMVs group. ATP‐citrate lyase (ACLY), a key enzyme in acetyl‐coenzyme A (CoA) synthesis, has attracted our attention. A recent study has shown that ACLY is essential for the establishment and maintenance of the pro‐inflammatory senescence‐associated secretory phenotype (SASP).^[^ ^27^ ^]^ Thus, we performed the ACLY immunofluorescence staining to evaluate its level in diabetic wounds. The results revealed that ACLY expression was significantly down‐regulated in all treatment groups, with the most pronounced reduction observed in the PVA‐MN@euMVs group (Figure S9, Supporting Information). This finding suggests that ACLY may be involved in mediating the inhibition and rescue of cellular senescence by Mito@euMVs. Moreover, protein‒protein interaction network analysis revealed that Mito@euMVs not only impacted proteins related to antioxidative stress, energy metabolism, and cell senescence but also demonstrated close interactions among these proteins (Figure S8C, D, Supporting Information). These results, consistent with the in vivo histochemical staining findings (Figures 7 and 8), provide further evidence that Mito@euMVs accelerated wound healing by enhancing mitochondrial function, energy metabolism, and antioxidative capacity while inhibiting cellular senescence in tissues.

## 3 Discussion

In this study, we prepared artificial microvesicles encapsulating active mitochondria from enucleated MSCs and investigated their efficacy in regulating cellular senescence. Our results demonstrated that the obtained Mito@euMVs can effectively deliver the enclosed mitochondria to recipient cells, inhibiting and reversing the hyperglycemia‐induced senescent phenotypes of both fibroblasts and HUVECs. Following the validation of the favorable impact of Mito@euMVs on cellular rejuvenation, we further loaded Mito@euMVs on soluble PVA‐MN patches to facilitate their application in a diabetic rat pressure sore model. PVA‐MN@euMVs showed a significantly enhanced pro‐healing capacity, and this noteworthy improvement may be mainly attributed to the senescence‐inhibiting and ‐rescuing mechanism achieved by the transfer of mitochondria through Mito@euMVs.

It has been reported that extracellular vesicles (EVs) secreted by MSCs naturally incorporate mitochondria during their biogenesis, making them suitable carriers for delivering mitochondria.^[^ ^28^ ^]^ The protection of the EVs membrane keeps the integrity and functional activity of the mitochondria intact. However, EVs‐mediated mitochondrial transfer suffers from the challenge of obtaining sufficient quantities of EVs containing mitochondria. The yield of EVs is quite low, and mitochondria (approximately 500–1000 nm in size) are predominantly present in medium‐to‐larger extracellular vesicles (m/lEVs, >200 nm in size),^[^ ^29^ ^]^ resulting in a further reduction in the yield of EVs containing mitochondria. A number of approaches, such as priming MSCs with growth factors and cytokines^[^ ^30^ ^]^ and genetically engineering MSCs,^[^ ^31^ ^]^ may increase EVs yield and mitochondrial load, but these approaches are often time‐consuming and expensive. In this study, by simply extruding the enucleated MSCs through the 3 µm‐sized membranes, we successfully obtained mitochondrial loaded microvesicles and significantly increased both the mitochondrial encapsulation efficiency and yield.

Recently, artificial nanovesicles prepared from MSCs through an extrusion approach have been rationally designed to mimic the functionalities and physicochemical properties of exosomes.^[^ ^32^ ^]^ This strategy aims to overcome the difficulties in the large‐scale and cost‐effective production of clinical‐grade exosomes, a challenge encountered in the clinical translation of exosome‐based therapies. The efficacy of these exosome‐mimicking nanovesicles is mainly attributed to the enclosed bioactive and therapeutic molecules, such as growth factors and anti‐inflammatory cytokines.^[^ ^32b^ ^]^ Similar to previously reported nanovesicles, Mito@euMVs contained pro‐healing factors from parental MSCs, contributing to the observed improvement in diabetic wound healing. Moreover, compared with exosomes and other nanovesicles, Mito@euMVs possess two distinct features. First, by controlling the vesicle size within the micron‐scale range (1‐4 µm), we ensured the encapsulation of MSCs mitochondria in Mito@euMVs, further endowing Mito@euMVs with the ability to deliver mitochondria and subsequently rescue cellular senescence. Another feature of Mito@euMVs lies in the reduction of nuclear materials. By removing the nuclei from MSCs before vesicle preparation, we effectively minimized the amount of nuclear material in the resulting microvesicles, thereby bolstering the safety profile of Mito@euMVs. While our validation efforts focused solely on elucidating the rescuing effects of Mito@euMVs on hyperglycemia‐induced senescent fibroblasts and HUVECs in vitro, as well as on diabetic wounds in vivo, we believe that Mito@euMVs, which serve as versatile mitochondria delivery vehicles, hold significant potential in a variety of diseases associated with mitochondrial dysfunction and cellular aging.

Although the present study has yielded exciting findings, it is imperative to acknowledge several limitations, necessitating further research to optimize this system. First, the quantity of mitochondria in each euMV represents a critical parameter for quality control. However, it is currently difficult to either characterize or precisely control the specific number of mitochondria contained in a single euMV. Second, although it has been reported that cell‐derived microvesicles are internalized via several mechanisms, including endocytosis, phagocytosis, and macropinocytosis, the specific mechanisms underlying the cellular uptake of Mito@euMVs remain unclear and require further research. Third, while Mito@euMVs exhibited impressive stability at ‐80°C, preserving mitochondrial activity is a significant challenge. Thus, it is desirable to develop novel approaches to prolong mitochondrial activity to facilitate the clinical translation of Mito@euMVs. Fourth, enhancing mitochondrial loading efficiency could be achieved through genetic modification of parental MSCs to stimulate mitochondrial biogenesis, such as the introduction of the peroxisome proliferator‐activated receptor γ‐coactivator 1‐α (PGC1‐α) gene into MSCs. Moreover, engineering targeting molecules on Mito@euMVs can confer the ability to target specific organs or recipient cells through intravenous injection, further expanding the potential applications of Mito@euMVs.

## 4 Experimental Section

### Cell Culture

Rat MSCs were isolated from the adipose tissue of six‐week‐old male rats and cultured with alpha‐minimum essential medium (𝛼‐MEM, Gibco, USA) supplemented with 10% fetal bovine serum (FBS, SORFA, China) and 1% penicillin/streptomycin (P/S, Gibco, USA) in a humidified atmosphere of 5% CO2 at 37°C. Rat dermal fibroblasts were isolated from the abdominal dermal tissue of six‐week‐old male rats. Human umbilical vein endothelial cells (HUVECs) were purchased from Procell (Wuhan, China). The healthy fibroblasts/HUVECs (Control) were cultured with low‐glucose Dulbecco's modified Eagle's medium (DMEM, Gibco, USA) supplemented with 10% fetal bovine serum (FBS, SORFA, China) and 1% P/S in a humidified atmosphere of 5% CO2 at 37°C. Senescent fibroblasts and HUVECs (Sen‐Control) were cultured in low‐glucose DMEM supplemented with 10% FBS, 1% P/S, and additional glucose to achieve a glucose concentration of 35 mM.

### Preparation and Characterization of Enucleated MSCs‐Derived Mito@euMVs

Adherent MSCs were digested with trypsin (Gibco, USA) and resuspended in α‐MEM at a density of 1×10^6^ cells mL^−1^. Cytochalasin B (CB, 1 mg mL^−1^, Aladdin, China) dissolved in DMSO (Aladdin, China) was added to the cell suspension to a final concentration of 10 µg mL^−1^. The cells were incubated in a cell incubator at 37°C for 2 h, centrifuged at 1200 rpm for 5 min, and then resuspended in 50% Percoll (Beyotime, China) solution containing 10 µg mL^−1^ CB. The suspension was centrifuged at room temperature at 21 000×g for 2 h, and the white membrane between the upper and lower layers was extracted and then resuspended in 4 volumes of cold α‐MEM. After centrifugation at 1000×g for 15 min, the sediment obtained was collected from the enucleated MSCs. The membranes of the enucleated cells were labeled with either 1,1'‐Dioctadecyl‐3,3,3',3'‐Tetramethylindodicarbocyanine,4‐Chlorobenzenesulfonate Salt (DiD) (Beyotime, China) or 1,1'‐dioctadecyl‐3,3,3',3'‐tetramethylindocarbocyanine perchlorate (DiI) (Beyotime, China), the nuclei were labeled with Hoechst 33342, and the intracellular mitochondria were labeled with Mito‐Tracker Green (Beyotime, China). Then, the enucleation efficiency of the cells was measured by flow cytometry. To prepare the MVs, the enucleated MSCs were suspended in PBS and extruded through different diameter micro/nanopore‐sized membranes (Whatman, UK) using a liposome extruder. Finally, MVs with diameters of 0.4 µm, 1.0 µm, and 3.0 µm were prepared. The MVs were precipitated by centrifugation at 21 000×g for 15 min. Subsequently, the sediment was resuspended in PBS for further experiments. The mitochondria in MVs with different particle sizes were detected by fluorescence imaging and flow cytometry. The morphology of the Mito@euMVs was visualized via High Intelligent and Sensitive Structured Illumination Microscope (HIS‐SIM, CRS Biotech, China) and transmission electron microscopy (Hitachi, Japan). The particle size distribution of the extruded Mito@euMVs was verified by flow cytometry using a flow cytometry size calibration kit (Thermo Fisher, USA). The zeta potential of the Mito@euMVs was detected by a Zetasizer Nano ZSE (Malvern, UK). After labeling the active mitochondria in the Mito@euMVs with tetramethylrhodamine methyl ester (TMRM), the stability of mitochondrial activity in the Mito@euMVs was characterized by flow cytometry at different time points.

### Isolation of Naked Mitochondria

Mitochondria were freshly isolated from rat adipose‐tissue derived MSCs using a mitochondrial isolation kit (Beyotime, China, C3601) following the manufacturer's instructions. Briefly, after trypsinization, cells were resuspended in 1 mL of cell lysis reagent and incubated on ice for 15 min. The lysed cells were then homogenized with a glass homogenizer for 30 cycles and centrifuged at 600 × g for 10 min at 4°C. The supernatant was collected and further centrifuged at 11 000 × g for 10 min at 4°C. The resulting pellet contained the isolated mitochondria. The activity of naked mitochondrial was monitored by TMRM staining using flow cytometry at different time points.

### Cellular SA‐β‐Gal Detection

The senescent state of fibroblasts and HUVECs was examined by evaluating the intracellular expression of senescence‐associated β‐Gal with a Senescence β‐Galactosidase Staining Kit (SA‐β‐Gal, Beyotime, China) following the manufacturer's instructions. The proportion of SA‐β‐Gal‐positive cells was calculated. Senescent fibroblasts and HUVECs (Sen‐Control) were cultured in low‐glucose DMEM supplemented with 10% FBS, 1% P/S, and additional glucose to achieve a glucose concentration of 35 mM. After senescent fibroblasts and HUVECs were treated with 20 µg mL^−1^ and 40 µg mL^−1^ Mito@euMVs or naked mitochondria for 0 and 5 days, SA‐β‐Gal was used to stain the cells, and the proportion of blue SA‐β‐Gal‐positive cells in each group was calculated. For the rotenone experiment, 1.5 µM rotenone (Aladdin, China) was added on day 0 and incubated with 20 µg mL^−1^ and 40 µg mL^−1^ Mito@euMVs for 5 days. SA‐β‐Gal was used to stain the cells, and the proportion of blue. SA‐β‐Gal‐positive cells in each group were calculated.

### ELISA

After treating the cells with the Mito@euMVs, the cell culture supernatant was collected and centrifuged at 1000 rcf at 4°C for 10 min to remove impurities such as cell debris. The levels of IL‐6 (E‐EL‐R0015c, Elabscience, China) and TNF‐α (E‐EL‐R2856c, Elabscience, China) in the culture media were measured and analyzed according to the instructions of the ELISA kit.

### RNA Isolation and Quantitative Reverse Transcription‑PCR

After treatment with the Mito@euMVs for 48 h, total RNA from the fibroblasts/HUVECs was isolated using a Total RNA Extraction Kit (Solarbio, China) according to the manufacturer's instructions and quantified with a NanoDrop 2000 spectrophotometer (Thermo Scientific, USA). The isolated RNA was then reverse‐transcribed into complementary DNA (cDNA) using a Prime Script RT reagent kit with gDNA Eraser (TaKaRa, Japan).

Quantitative real‐time PCR was performed using the All‐in‐One SYBR Green system q‐PCR Mix (GeneCopoeia, USA) on a 96‐well real‐time PCR device (LightCycler® 96 Roche, Swiss). The fold change in the expression of each target gene was normalized to that of the housekeeping gene glyceraldehyde‐3‐phosphate dehydrogenase (GAPDH) and calculated using the 2^−ΔΔCt^ method. The sequences of primers used in this study are shown in Table S1 (Supporting Information).

### Characterization of Mitochondrial Transfer

After cells were seeded into confocal dishes, the mitochondrial networks in the cells were labeled with PK Mito Deep Red (Nanjing Warbio, China). Meanwhile, the mitochondria enclosed in the euMVs were labeled with PK Mito Red (Nanjing Warbio, China). After coculturing the Mito@euMVs with cells for 24 h, the nuclei of the cells were labeled with Hoechst 33342, and the autophagosomes were stained with an Autophagy Staining Assay Kit with MDC (Beyotime, China), and the fusion of the mitochondria in the Mito@euMVs with the mitochondrial network of the fibroblasts and HUVECs was imaged by HIS‐SIM (CRS Biotech, China).

### Mitochondrial Membrane Potential in Cells

After cells were treated with either Mito@euMVs or naked mitochondria for 48 h, the mitochondria in the cells were labeled with TMRM for 30 min in a cell incubator at 37°C for 30 min. After being washed twice with PBS, the nuclei of the cells were stained with Hoechst 33342 at 37°C for 10 min and washed twice with PBS. The TMRM‐labeled mitochondrial network was photographed by a confocal microscope under the same imaging conditions. Flow cytometry was conducted to quantitatively assess the fluorescence intensity in individual cells, analyzing 5000 cells per sample.

### Antioxidant Efficacy of Mito@euMVs on Senescent Cells

Non‐senescent and senescent cells were seeded into 24‐well plates at a density of 2×10^4^ cells per well. After overnight incubation, either Mito@euMVs or naked mitochondria were added to the medium of senescent cells and co‐incubated for 48 h. To assess the production of ROS in cells, a ROS assay kit (Beyotime, China) was used to detect intracellular ROS. Briefly, the cells were incubated with 5 µM DCFH‐DA at 37°C for 30 min in the dark. Fluorescence images were acquired via laser confocal microscopy. Flow cytometry was conducted to quantitatively assess the fluorescence intensity in individual cells, analyzing 5000 cells per sample.

### ATP Production and the ADP/ATP Ratio of Cells

Non‐senescent and senescent cells were seeded in 24‐well plates (Corning, USA) at a density of 2×10^4^ cells per well, and the senescent cells were treated with 20 µg mL^−1^ and 40 µg mL^−1^ Mito@euMVs respectively for 48 h. ATP production was detected with an ATP assay kit according to the manufacturer's instructions (Beyotime, China). In brief, ATP concentration gradient solutions corresponding to the standard curve were prepared according to the kit instructions. After the cell culture medium was removed, 200 µL of cell lysate was added to each well, and all the liquid was transferred to an Eppendorf (EP) tube and centrifuged at 12000 rcf at 4°C for 5 min. Then, 100 µL of working solution was added to a black 96‐well plate, and after the plate was incubated at room temperature for 3–5 min, 20 µL of cell lysate or ATP standard solution was added to each well to measure the chemiluminescence. The remaining lysate in each well was measured with a BCA protein assay kit (Beyotime, China) for normalization of ATP production.

To determine the ADP/ATP ratio, non‐senescent and senescent cells were seeded in white 96‐well plates (Corning, USA) at a density of 2000 cells per well, and the senescent cells were treated with 20 µg mL^−1^ and 40 µg mL^−1^ of Mito@euMVs respectively for 48 h. The ADP/ATP ratio of cells was detected by an ADP/ATP ratio assay kit according to the manufacturer's instructions (Dojindo, Japan). In brief, after the cell culture medium was removed, 90 µL of ATP working solution was added to each well, and the cell culture medium was shaken in a multifunctional microplate reader (Tecan, Switzerland) for 2 min and incubated at 25°C for 10 min. The chemiluminescence Data1 value was detected. ADP working solution was prepared, and the chemiluminescence value (Data2) was detected before 5 µL of ADP working solution was added to each well. After shaking for 2 min, the mixture was incubated at 25°C for 8 min, after which the chemiluminescence value (Data 3) was detected. The ADP/ATP ratio was calculated according to the following formula:

(1) $$ADP/ATP=\left(Data3-Data2\right)/Data1$$

### Preparation and Characterization of PVA‐MN Patches

15%, 20%, and 25% PVA solutions were prepared by dissolving 1.5 g, 2.0 g, and 2.5 g of 1788PVA (Aladdin, China), respectively, in 10 mL of dH_2_O solution at 90°C for 4 h until completely dissolved. After cooling to room temperature, 20 µL of the above solution was poured over a polydimethylsiloxane (PDMS, Corning, USA) mold containing an array of MN cavities. The PDMS model was placed in a vacuum environment at room temperature for 30 min to remove air bubbles at the bottom of the MN mold. Then, the mold was placed in a drying oven at 40°C overnight. Finally, the MN patch was detached from the PDMS mold. The mechanical properties of the MN patches were characterized by a universal testing machine (Instron, USA) and a porcine skin puncture experiment. The dissolution rate of the tip in vitro was verified by inserting the MN sheet into a 15% GelMA hydrogel. To prepare PVA‐MNs loaded with Mito@euMVs, Mito@euMVs were added to cooled PVA solution at a final concentration of 10 µg µL^−1^ and thoroughly mixed. Subsequently, 20 µL of solution containing Mito@euMVs was dispensed into the PDMS mold, the excess solution was recovered, and the top of the mold was filled with PVA solution without Mito@euMVs and dried at 40°C overnight. To evaluate the stability of the Mito@euMVs and the activity of the mitochondria enclosed within the PVA‐MN patch, 3D images of the MN patch were captured via confocal microscopy at different time points subsequent to labeling the Mito@euMVs membrane with DiI and mitochondria within it with Mito‐Tracker Green.

### Animals

All animal procedures were approved by the Animal Care and Use Committee of South China University of Technology (Ethics Number: 2022106). Three‐week‐old male Sprague‒Dawley rats were purchased from the Experimental Animal Center of SCUT, Guangzhou. In vivo wound healing was investigated with a streptozotocin‐induced diabetic wound model. After the rats were anesthetized with isoflurane (RWD, China), the back hair was removed with a shaving knife and hair removal cream. The skin on the rats’ backs was pinched and secured on both sides using a pair of circular magnets with a diameter of 20 mm. After 24 hours, the magnets were removed, and two pressure sore wounds formed on the back. The rats were randomly divided into four groups: Control (no treatment), PVA‐MN (pure PVA‐MN), ID@MVs (Mito@euMVs injected intradermally), and PVA‐MN@MVs (Mito@euMVs loaded in PVA‐MN). The wounds were subjected to different treatments on days 0, 1, and 2 after wound formation. Specifically, the wounds in the Control group did not receive any treatment; the wounds in the PVA‐MN group were treated with euMV‐free PVA‐MN patches at four locations around the edge; the wounds in the ID@MVs group were treated with 20 µg per injection point for a total of 4 injection points; and the wounds in the PVA‐MN@euMVs group were treated with the Mito@euMVs‐loaded PVA‐MN patches at four locations around the edge. The wounds were photographed and analyzed by ImageJ software on days 0, 3, 7, 14, and 21. The wound tissues, along with surrounding areas, were harvested on days 7, 14, and 21 for further histological analysis after the rats were euthanized using a carbon dioxide smothering machine. n = 6 for each treatment group at each time point.

### Histological Analysis

For the paraffin sections, the fresh skin tissues were immediately fixed in 4% paraformaldehyde at 4°C overnight. The samples were then dehydrated in a progressive manner and embedded in paraffin before sectioning into 6‐µm slices for further H&E staining, Masson trichrome staining, immunohistochemical staining, and multiplex immunofluorescence staining.

For multiplex immunofluorescence (mIHC) staining, paraffin sections were baked for 12 h at 60°C, dewaxed with xylene, and rehydrated with graded alcohol. After rehydration, the sections were stained using mIHC kits (#NEL861001KT, AKOYA Biosciences, USA) following the manufacturer's instructions. Scanning images of tissue samples were obtained using an Akoya Phenocycler Fusion (AKOYA Biosciences, USA), and the images were processed using Phenochart (version 1.2.0) and inForm (version 2.6.0). The fluorescence images were imported into the open‐source software platform QuPath, and the cell detection step was performed. The StarDist cell segmentation script (.groovy file) was dragged and dropped into the QuPath window. Object classification was set, and the object classifier was trained based on cell fluorescence intensity. After training the classifier, it was assigned a name and saved. The same classifier was then used for cell segmentation and data analysis across different groups. Antibodies were used according to the recommended antigen retrieval solution, dilution and incubation time, and their details are provided in Table S2 (Supporting Information).

For frozen sections, fresh skin tissue was immediately frozen with liquid nitrogen. After being embedded in OCT (SAKURA, USA), the samples were sectioned into 10‐µm slices with a freezing microtome (Leica, Germany) for further SA‐β‐Gal staining, immunofluorescence staining, and DHE staining.

For SA‐β‐Gal staining, frozen sections of fresh tissue were washed 3 times in PBS to remove excess OCT, and the tissue was collected with an IHC pen (Biosharp, China). After preparing the SA‐β‐Gal staining working solution according to the instructions of the kit, the solution was gently dripped on the tissue and incubated in a wet box in the dark at 37°C in a carbon dioxide‐free environment overnight. After sealing, the slices were photographed under a light microscope, and the proportion of the blue area in each group was determined.

For immunofluorescence staining, frozen sections of fresh tissue were fixed in cold acetone for 10 min and washed three times with PBS, and the tissue was collected with an IHC pen. The tissues were treated with 0.1% Triton‐PBS (Beyotime, China) for 10 min and then washed twice with PBS. After the tissue was blocked with 5% BSA (VETEC, Germany) for 45 min at room temperature, the blocking solution was removed, and the primary antibody solution diluted according to the antibody manufacturer's instructions was added to the tissue and incubated overnight in a wet box at 4°C in the dark. The primary antibody solution was removed by washing three times with PBS, and a fluorescent secondary antibody solution diluted (1:200) in PBS was added and incubated for 2 h at room temperature. Unbound secondary antibodies were removed by washing three times with PBS. The tissues were stained with DAPI for 5 min at room temperature to label the nuclei of the cells, washed three times with PBS and sealed, and tissue sections from each group were photographed with an inverted fluorescence microscope (Nikon, Japan).

For DHE staining, frozen sections of fresh tissue were washed 3 times in PBS to remove excess OCT, and the tissue was collected with an IHC pen (Biosharp, China). DHE (5 µM; Beyotime, China) solution was gently added to the tissue and incubated in a wet box in the dark at 37°C for 30 min. The DHE solution was removed by washing three times with PBS. The tissues were stained with DAPI for 5 min at room temperature to label the nuclei of the cells, washed three times with PBS and sealed, and tissue sections from each group were photographed with an inverted fluorescence microscope.

### Proteomic Analysis

To examine the differentially expressed proteins (DEPs) in Control, PVA‐MN, ID@euMVs and PVA‐MN@euMVs, proteomics was commercially commissioned to Suzhou PANOMlX Biomedical Tech Co., Ltd. (Suzhou, China) for total protein qualitative and relative quantitative analysis using label‐free full proteomics. Based on the Gene Ontology database, the protein signatures were functionally annotated using the “function annotation” module of the DAVID website. GseaVis packages in R software or the SangerBox website. Subcellular localization analysis of the proteins was performed using the WoLF PSORT website.

Proteins with a fold change ≥ 1.5/fold change ≤ 0.67 and p value ≤ 0.05 were considered significantly differentially expressed proteins (DEPs). A fold change (FC) ≥ 1.5 represented upregulated proteins, while a FC ≤ 0.67 represented downregulated proteins. The p‐value was calculated based on the peptide segments using one‐way ANOVA.

### Statistical Analysis

Independent experiments with at least three replicates per group were performed three times to assure repeatability (n ≥ 3). The statistical significance of differences between two groups was determined using an independent unpaired two‐tailed Student's t test. Comparisons among more than two groups were analyzed using one‐way ANOVA followed by Tukey's test. The minimum significance level was set at *p < 0.05, **p < 0.01, ***p < 0.001, and ****p < 0.0001.

## Conflict of Interest

The authors declare no conflict of interest.

## Author Contributions

Z.D. and X.L. contributed equally to this work. Conceptualization: X.F. Data curation: Z.D. and X.L.; Formal analysis: X.L. and Z.D.; Funding acquisition: X.F.; Investigation: X.L. and Z.D.; Methodology: X.F. and X.L.; Project administration: X.F. and X.L.; Software: S.L., Z.D., and X.L.; Visualization: Z.D., X.L., and S.L.; Writing – original draft: X.L. and Z.D.; Writing – review & editing: X.F. and Z.D.

## Supporting information



Supporting Information

## Data Availability

The data that support the findings of this study are available from the corresponding author upon reasonable request.
