# Supplementary material for: Inhibition and Rescue of Hyperglycemia‐Induced Cellular Senescence by Mitochondrial Transfer from Enucleated Mesenchymal Stem Cell‐Derived Microvesicles for Chronic Wound Healing
Source: Adv Sci (Weinh). 2025 May 23;12(30):e01612. doi: 10.1002/advs.202501612 (PMC12376497; doi:10.1002/advs.202501612)
Supplement: Supplementary file 1 — Supporting Information [file ADVS-12-e01612-s001.docx]

**Supporting Information**

**Inhibition and Rescue of Hyperglycemia-Induced Cellular Senescence by Mitochondrial Transfer From Enucleated Mesenchymal Stem Cell-Derived Microvesicles for Chronic Wound Healing**

Zixuan Dong^1^, Xiaobing Liu^1^,Shichun Li^1^, Xiaoling Fu^1,2,3, *^

^1.^ School of Biomedical Sciences and Engineering, South China University of Technology, Guangzhou International Campus, Guangzhou 511442, P. R. China

^2.^ National Engineering Research Center for Tissue Restoration and Reconstruction and Innovation Center for Tissue Restoration and Reconstruction, Guangzhou 510006, P. R. China.

^3.^ Laboratory of Biomedical Engineering of Guangdong Province, South China University of Technology, Guangzhou 510006, P. R. China.

^*.^ Correspondence address. School of Biomedical Sciences and Engineering, South China University of Technology, Guangzhou 510006, P. R. China. Email: msxlfu@scut.edu.cn (Xiaoling Fu).

**Materials and Methods**

**Mito@euMVs and naked mitochondria uptake assay**

To detect the uptake of Mito@euMVs by cells *in vitro*, cells were seeded in confocal dishes at a density of 50,000 cells/dish (Biosharp, China). After 12 h, the Mito@euMVs were labeled with 1,1'-dioctadecyl-3,3,3',3'-tetramethylindocarbocyanine perchlorate (DiI) (Beyotime, China) according to the manufacturer’s instructions and added to cultured cells at concentrations of 20 μg/mL and 40 μg/mL. For naked mitochondria uptake, the naked mitochondria labeled with Mito-Tracker Red (Beyotime, China) were added to cultured cells at concentrations of 20 μg/mL and 40 μg/mL . After incubation for 24 h, the recipient cells and control cells were washed with phosphate-buffered saline (PBS) twice, fixed in 4% paraformaldehyde (PFA) for 30 min at room temperature, and then washed with PBS twice. Then, the nuclei were stained with 4',6-diamidino-2-phenylindole (DAPI) (Beyotime, China), and the F-actin of the cells was stained with YF®488-Phalloidin (US Everbright, China). Fluorescence images were acquired via confocal laser scanning microscopy (CLSM) (Zeiss, Germany). To evaluate the cell uptake efficiency, flow cytometry was conducted after staining.

**Fibroblast migration assay**

Non-senescent and senescent fibroblasts were seeded into six-well plates at a density of 5×10^5^ cells/well. When the cells reached 90% confluence, sterile 200 μL pipette tips were used to create scratches on the plates by scraping the cells. After washing with PBS twice, the medium was changed to culture medium without fetal bovine serum (FBS). Mito@euMVs (20 μg/mL and 40 μg/mL) dissolved in PBS were added to specific wells as indicated. Scratches were photographed at 0, 24, and 48 h with an inverted microscope (Nikon, Japan). Quantification of the scratch area was performed using ImageJ software (NIH, USA).

**Cell Counting Kit-8 analysis**

Cell proliferation was investigated by a Cell Counting Kit-8 (CCK-8) assay (APExBIO, USA). Briefly, non-senescent and senescent fibroblasts or HUVECs were seeded in 48-well plates at a density of 4 × 10^3^ cells/well. Mito@euMVs (20 μg/mL and 40 μg/mL) were added to the culture medium. 200 μL of culture medium and 20 µL of CCK-8 solution were added to each well on days 1, 3, and 5, and the plates were incubated at 37 °C for 1.5 h. The light absorbance was measured at 450 nm with a microplate reader (TECAN, Switzerland). The results are presented as the optical density (OD) values minus the absorbance of the blank wells.

**HUVEC tube formation assay**

The tube formation assay was performed by μ-Slide Angiogenesis (ibidi, Germany) with Matrigel® (Corning, USA). Briefly, 10 μL of Matrigel was applied to each inner well of the μ-Slide chamber, which was then placed into the incubator for 30 min to gelate. HUVECs and HUVECs co-incubated with 20 μg/mL and 40 μg/mL of Mito@euMVs respectively for 48 h in advance were suspended in DMEM to a final density of 2 × 10^5^ cells/mL, and 50 μL of cell suspension was added to the upper well of the chamber. The samples were observed with a microscope (Nikon) at distinct time points. The total length of the tubes was calculated using the Angiogenesis Analyze plugin of ImageJ.

**Transwell migration assay**

A transwell migration assay of HUVECs was conducted in 24-well plates with transwell inserts of 8 μm pore size (Corning, USA) according to the manufacturer’s instructions. Briefly, HUVECs were resuspended in 200 μL of serum-free DMEM at a density of 1 × 10^5^ cells/mL and then seeded onto the upper chamber. Mito@euMVs (20 μg/mL or 40 μg/mL) in 600 μL of DMEM supplemented with 10% FBS were added to the lower chambers. The same volume of PBS was added to DMEM containing 10% FBS as a control. After 24 h, the chamber was removed, the medium in the upper chamber was removed, and the cells on the top layer of the membrane were gently removed with a cotton swab. The lower layer of cells was fixed with 4% PFA (Biosharp, China) at room temperature for 30 min, gently washed twice with PBS, and air-dried. After that, 400 μL of 0.1% crystal violet staining solution (Biosharp, China) was added to the well plate, and the lower layer of cells was stained for 10 min at room temperature. Excess dye was removed by washing with PBS twice. The samples were observed with a microscope to calculate the number of cells.

| **Human** | Forward (5’→3’) | Reverse (5’→3’) |
| --- | --- | --- |
| *GAPDH* | GCACCGTCAAGGCTGAGAAC | TGGTGAAGACGCCAGTGGA |
| *VEGF* | GTCCCATGAAGTGATCAAGTTC | TCTGCATGGTGATGTTGCTCTCTG |
| *eNOS* | AAGATCTCCGCCTCGCTCA | GCTGTTGAAGCGGATCTT |
| *bFGF* | GGCTTCTTCCTCCTGCGCATCCA | GCTCTTAGCAGACATTGGAAGA |
| *P16* | GCCCAACGCACCGAATAGTTAC | GCAGCAGCTCCGCCACTC |
| *P21* | GTCCAGCGACCTTCCTCATCC | CCATAGCCTCTACTGCCACCATC |
| **Rat** | Forward (5’→3’) | Reverse (5’→3’) |
| *GAPDH* | ATGGTGGTGAAGACGCCAGTA | GGCACAGTCAAGGCTGAGAATG |
| *ZBP1* | ATGGCGGAGGCTTCTGTG  GAC | CAGGACTACCAGCATCAC  TCAACAC |
| *P16* | CCTTGGCTTCACTTCTGGCAAC | CTCTGTCCCTCCCTCCCTCTG |
| *P21* | TAGGACTTCGGGGTCTCCTT | GCTCTGGACGGTACGCTTAG |
| *p53* | GTTCCGAGAGCTGAATGAGG | TTTTATGGCGGGACGTAGAC |
| *TGF-β* | ACCGCAACAACGCCATCTATGAG | GGCACTGCTTCCCGAATGTCTG |

**Table S1. The sequences of primers used in this study.**

**Table S2. mIHC Antibody Panel.**

| Antibody | Source | Cat# | Dilution | Incubation | Opal |
| --- | --- | --- | --- | --- | --- |
| CD68 | Abcam， USA | ab303565 | 1：250 | 1 h at RT | 480 |
| p53 | Abcam， USA | ab33889 | 1：100 | 2 h at RT | 570 |
| iNOS | Abcam， USA | ab283655 | 1：1000 | 12 h at 4℃ | 690 |
| Arg1 | CST, Germany | 93668 | 1：100 | 1 h at RT | 520 |
| Ki67 | Abcam， USA | ab16667 | 1：100 | 2 h at RT | 780 |


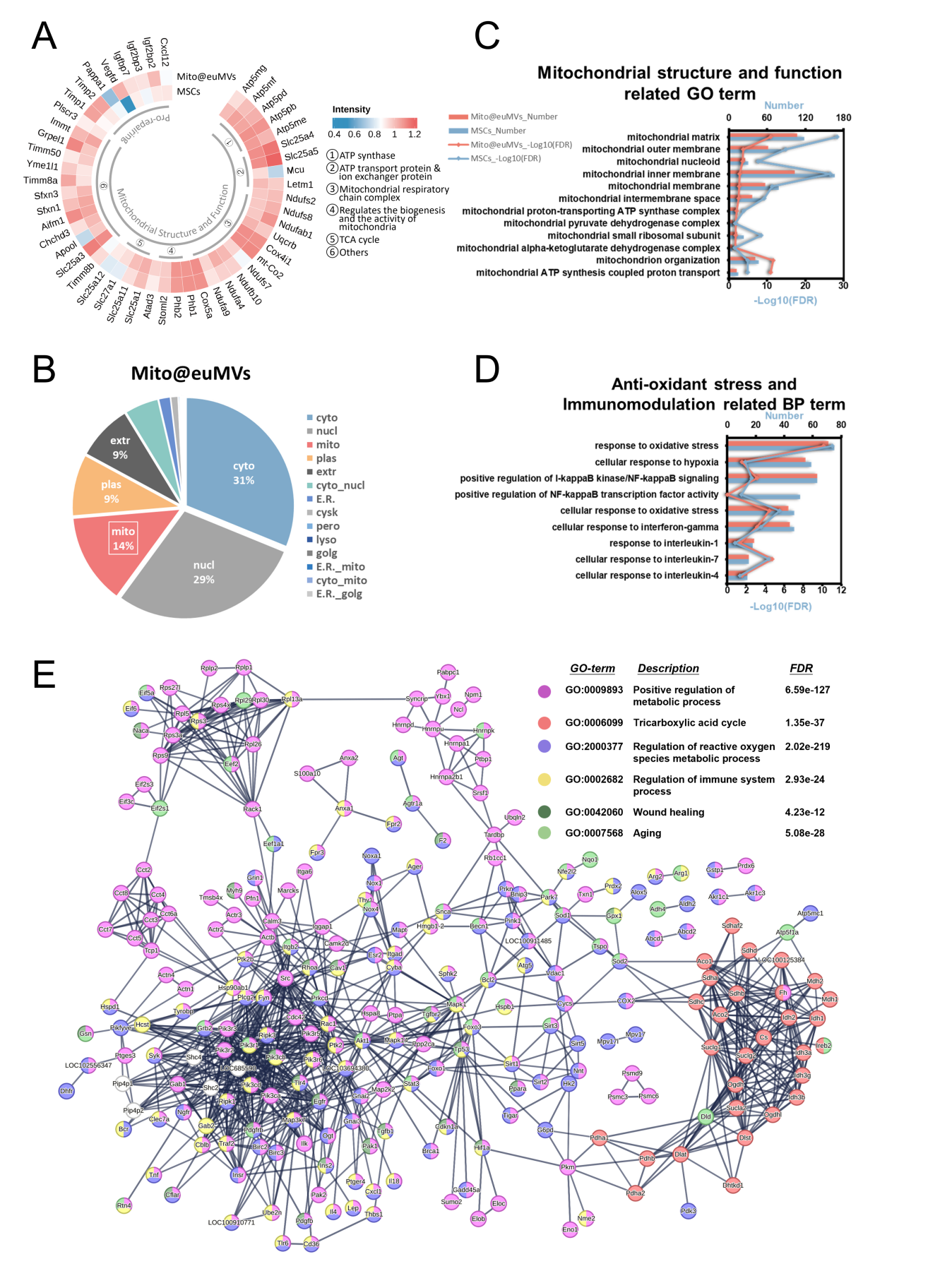


**Figure S1. Proteomic analysis of Mito@euMVs**

(A) Pro-healing proteins and mitochondrial proteins in Mito@euMVs and MSCs. (B) Subcellular localization analysis showing the intracellular distribution of all identified proteins in Mito@euMVs. (C-D) GO functional annotation analysis of the proteins in Mito@euMVs/MSCs. (E) PPI analysis of highly expressed proteins in Mito@euMVs. Nodes represent proteins, colors represent corresponding biological processes, and edges represent protein-protein interactions.


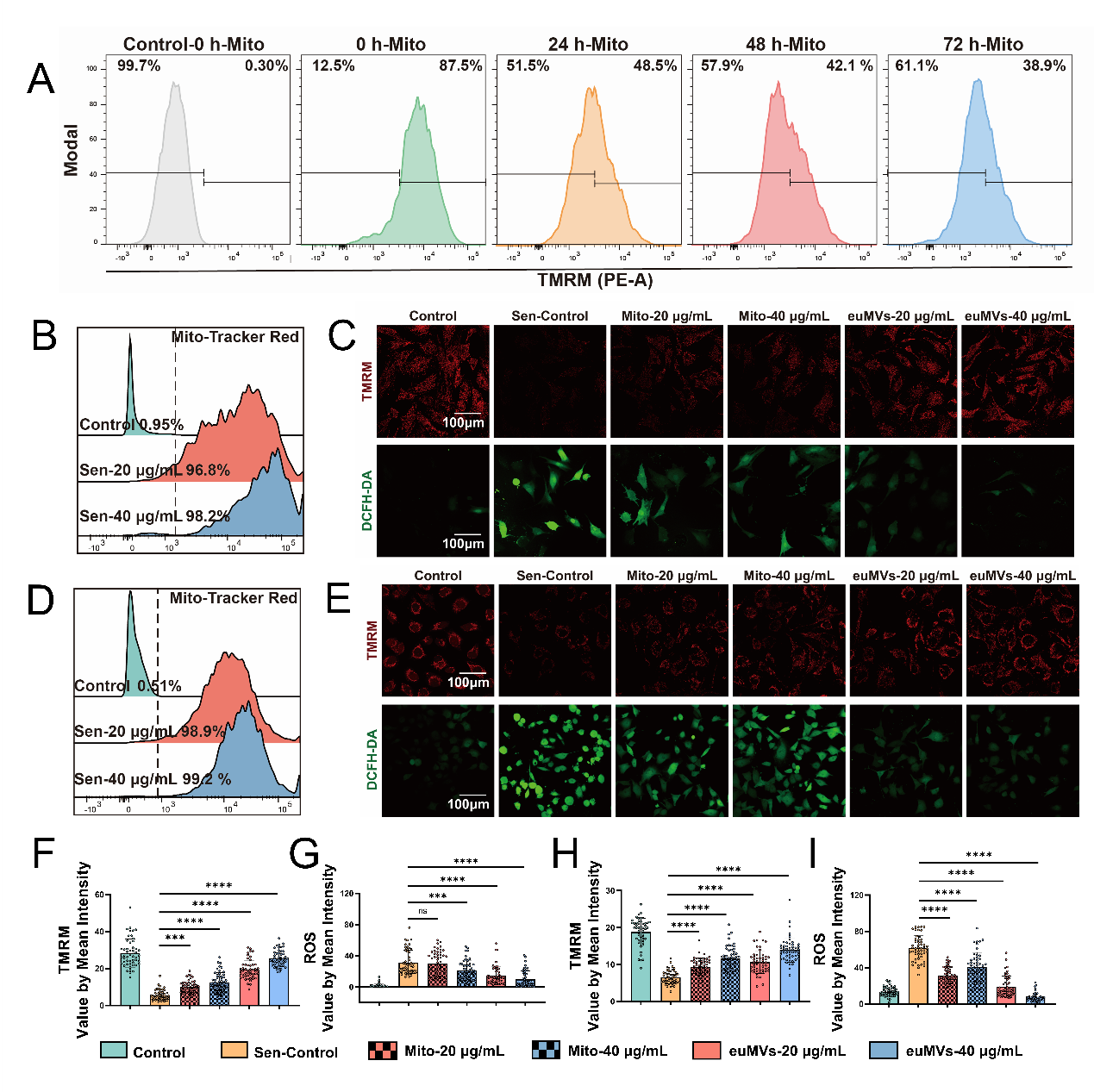


**Figure S2.** **Mito@euMVs exhibit superior efficacy in enhancing mitochondrial function in senescent fibroblasts and HUVECs compared to naked mitochondria isolated from MSCs.**

(A) Mitochondrial activity in naked mitochondria assessed by flow cytometry. Control-0 h-Mito refers to naked mitochondria that were not labeled with TMRM, whereas 0 h/24 h/48 h/72 h-Mito are naked mitochondria labeled with TMRM and analyzed at indicated time points. The data was analyzed in Flowj. (B) Uptake efficiency of naked mitochondria at various concentrations by senescent fibroblasts determined by flow cytometry. The Fibroblasts were treated with Mito-Tracker Red-labeled naked mitochondria for 24 h before analysis by flow cytometry. (C) Representative fluorescence images showing the mitochondria membrane potential of fibroblasts determined via TMRM staining (line 1) and intracellular ROS levels in fibroblasts detected by ROS probe DCFH-DA (line 2) with/without receiving treatment of Mito@euMVs (euMVs-20$\mu g$/mL and euMVs-40$\mu g/$mL) or naked mitochondria. (D)Uptake efficiency of naked mitochondria at various concentrations by senescent HUVECs determined by flow cytometry. The HUVECs were treated with Mito-Tracker Red-labeled naked mitochondria for 24 h before analysis by flow cytometry. (E) Representative fluorescence images showing the mitochondria membrane potential of HUVECs determined via TMRM staining (line 1) and intracellular ROS levels in HUVECs detected by ROS probe DCFH-DA (line 2) with/without receiving treatment of Mito@euMVs (euMVs-20$\mu g$/mL and euMVs-40$\mu g/$mL) or naked mitochondria. (F) The average fluorescence intensity of TMRM in individual fibroblasts analyzed using ImageJ. (G) The average fluorescence intensity of DCFH-DA in individual fibroblasts analyzed using ImageJ. (H) The average fluorescence intensity of TMRM in individual HUVECs analyzed using ImageJ. (I) The average fluorescence intensity of DCFH-DA in individual HUVECs analyzed using ImageJ. n=3, ***p<0.001, ****p<0.0001 vs Sen-Control. All data are presented as the mean ± SD.


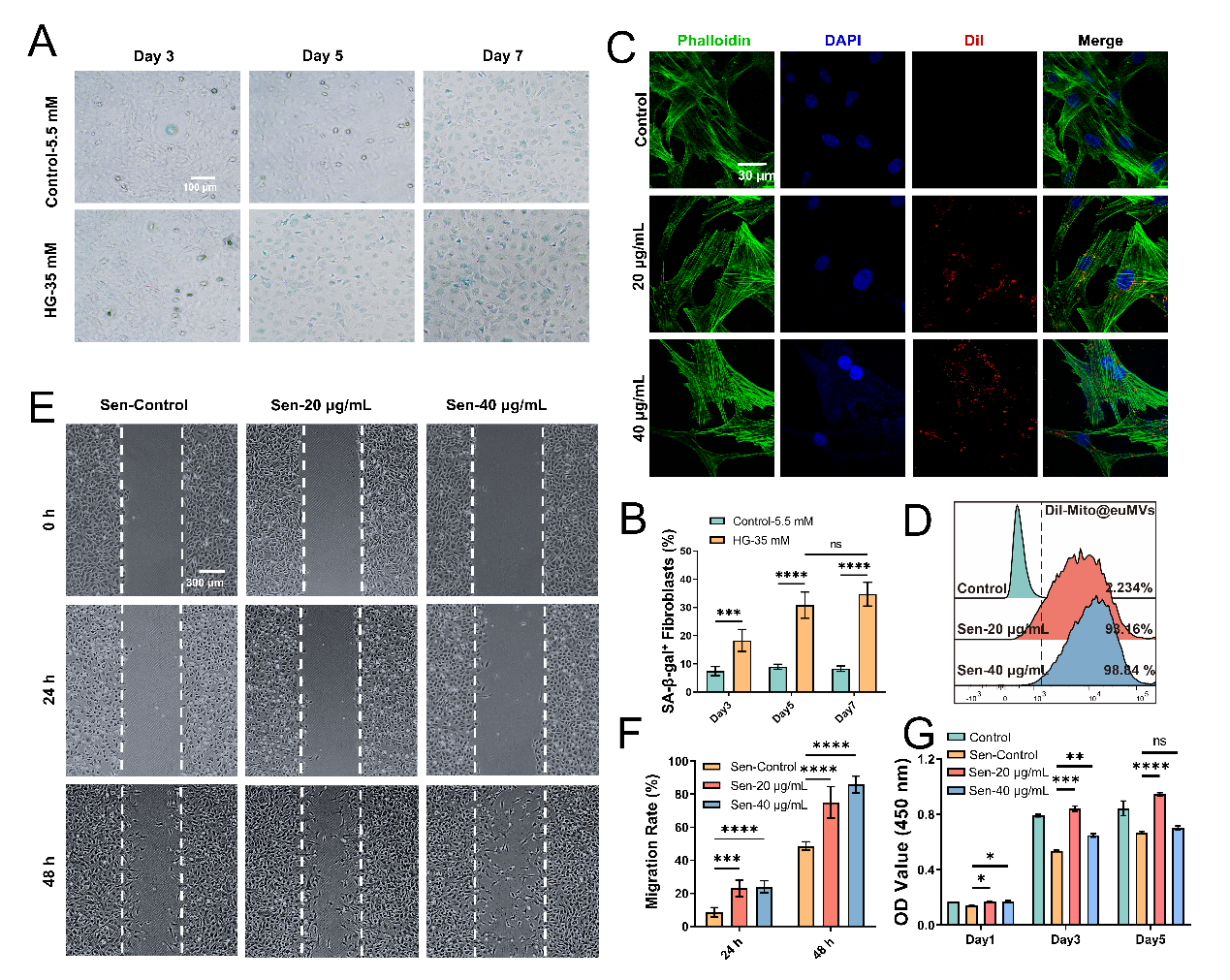


**Figure S3. Mito@euMVs improve the migration and proliferation activity of** **senescent fibroblasts**

(A) Representative bright-field images showing the senescent-associated beta-galactosidase (SA-β-Gal) expression in fibroblasts. Fibroblasts in the Control group were cultured in the medium with 5.5 mM glucose concentration, fibroblasts in the HG group were cultured in the medium with 35 mM glucose concentration. (B) The proportion of SA-β-Gal positive fibroblasts. ***p < 0.001, ****p<0.0001 vs Control-5.5 mM. (C) Representative fluorescence images showing the uptake of Mito@euMVs by senescent fibroblasts. The fibroblasts were treated with DiI-labeled Mito@euMVs for 24 h before images acquired via confocal microscopy. (D) Uptake efficiency of Mito@euMVs at various concentrations by senescent fibroblasts determined by flow cytometry. The fibroblasts were treated with DiI-labeled Mito@euMVs for 24 h before analysis by flow cytometry. (E) Representative bright-field images showing the migration of senescent fibroblasts treated by PBS (Control), 20 μg/mL and 40 μg/mL Mito@euMVs at indicated timepoints (0 h, 24 h, 48 h). (F) Quantification of migration rate of senescent fibroblasts. ***p < 0.001, ****p<0.0001 vs Sen-Control. (G) Cell viability of senescent fibroblasts, treated with Mito@euMVs for 1, 3, and 5 days, was measured by CCK-8. n=3, *p<0.05, **p<0.01, ***p < 0.001, ****p<0.0001 vs Sen-Control. All data are presented as the mean ± SD.


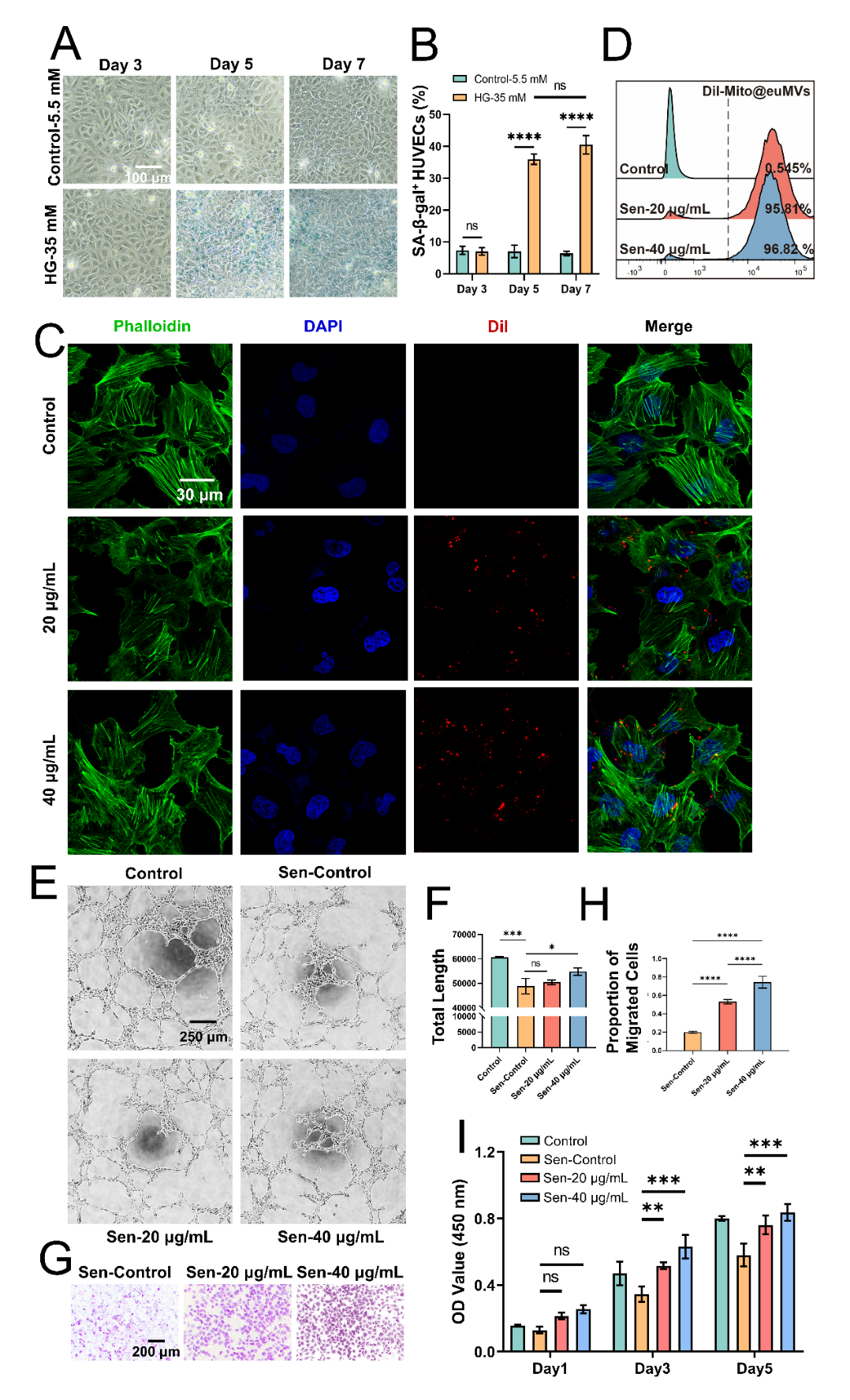


**Figure S4. Mito@euMVs improve the tube formation and migration of senescent HUVECs**

(A)Representative bright-field images showing the senescent-associated beta-galactosidase (SA-β-Gal) expression in HUVECs. HUVECs in the Control group were cultured in the medium with 5.5 mM glucose concentration, HUVECs in the HG group were cultured in the medium with 35 mM glucose concentration. (B) The proportion of SA-β-Gal positive HUVECs. ****p<0.0001 vs Control-5.5 mM. (C) Representative fluorescence images showing the uptake of Mito@euMVs by senescent HUVECs. The HUVECs were treated with DiI-labeled Mito@euMVs for 24 h before images acquired via confocal microscopy. (D)Uptake efficiency of Mito@euMVs at various concentrations by senescent HUVECs determined by flow cytometry. The HUVECs were treated with DiI-labeled Mito@euMVs for 24 h before analysis by flow cytometry (E) Representative bright-field images showing tube formation of HUVECs. (F) Analysis of tube formation total length.(G) Representative bright-field images showing the migrated senescent HUVECs in transwell. (H) Analysis of migrated senescent HUVECs proportion. (I) Cell viability of HUVECs, treated with Mito@euMVs for 1, 3, and 5 days, was measured by CCK-8. n=3, *p<0.05, **p<0.01, ***p < 0.001, ****p<0.0001 vs Sen-Control. All data are presented as the mean ± SD.


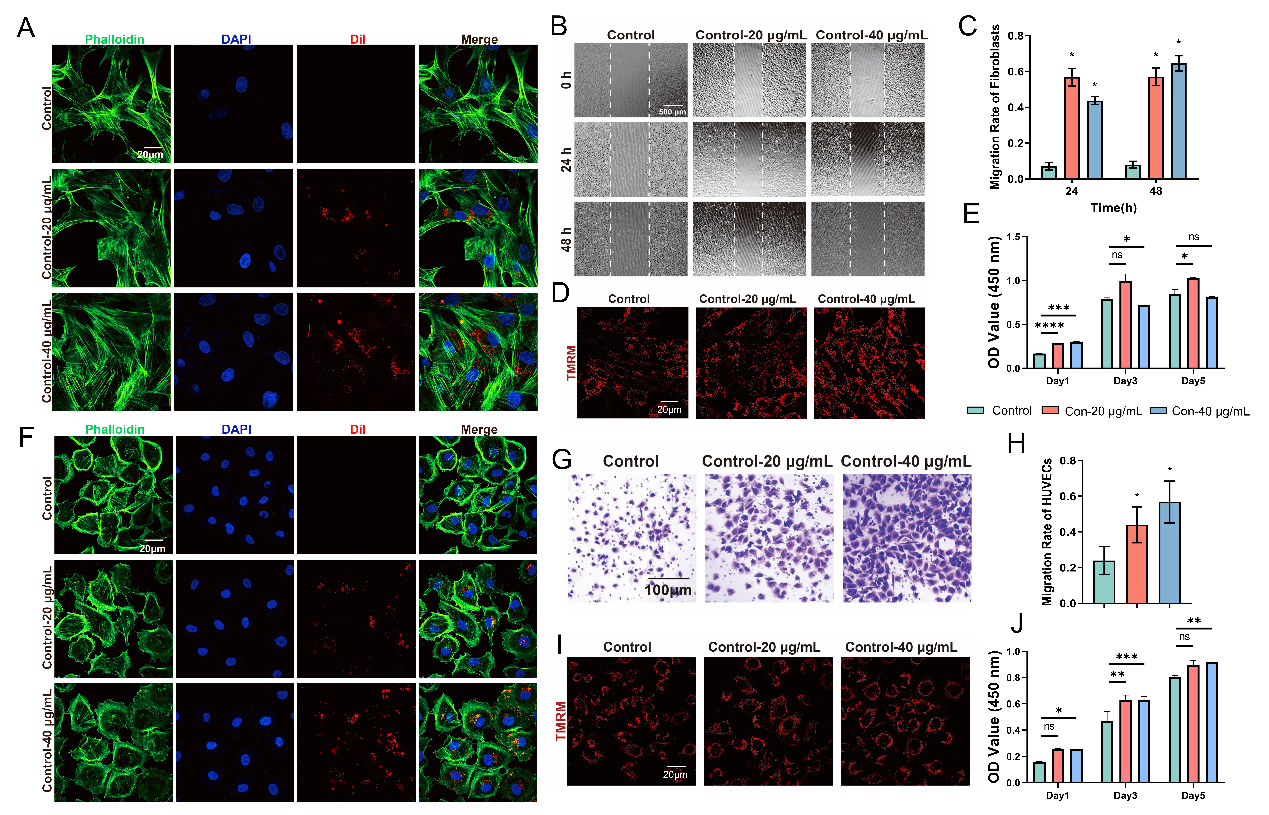


**Figure S5. Regulation of Mito@euMVs on non-senescent fibroblasts and HUVECs**

(A) Representative fluorescence images showing the uptaking of Mito@euMVs by non-senescent fibroblasts. (B) Representative bright-field images showing the migration of non-senescent fibroblasts at indicated time points (0 h, 24 h, 48 h). (C) Quantification of migration rate of fibroblasts. (D) Representative fluorescence images showing the mitochondria membrane potential of non-senescent fibroblasts determined via TMRM staining. (E) Cell viability of fibroblasts measured by CCK-8. (F) Representative fluorescence images showing the uptaking of Mito@euMVs by non-senescent HUVECs. (G) Representative bright-field images showing the migrated non-senescent HUVECs in transwell. (H) Quantification of migration rate of HUVECs. (I) Representative fluorescence images showing the mitochondria membrane potential of non-senescent HUVECs determined via TMRM staining. (J) Cell viability of HUVECs measured by CCK-8. n=3, *p<0.05, **p<0.01, ***p < 0.001, ****p<0.0001 vs -Control. All data are presented as the mean ± SD.


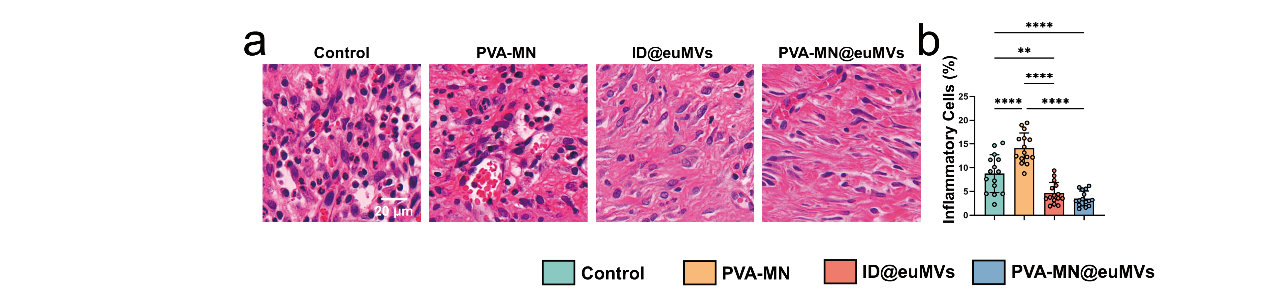


**Figure S6. Inflammation analysis of tissue on day 21.**

(A) H&E staining of wounds on day 21. (B) Proportion of inflammatory cell infiltration. n=6, **p<0.01, ****p<0.0001. All data are presented as the mean ± SD.


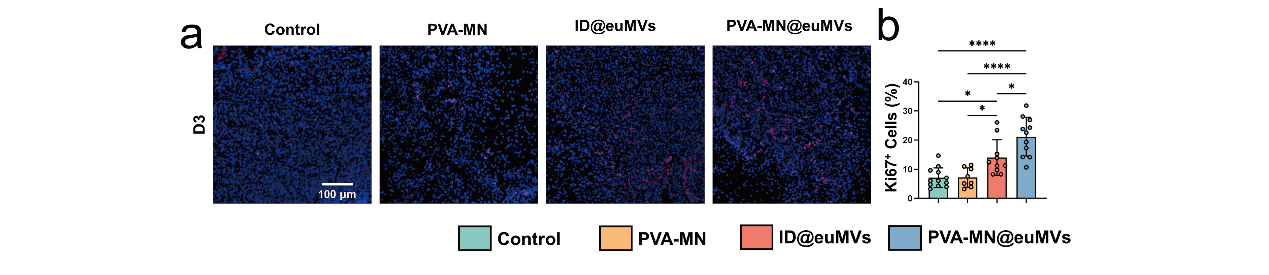


**Figure S7. ID@euMVs and PVA-MN@euMVs stimulate cell proliferation in diabetic pressure sores in rats.**

(A-B) Representative immunohistochemical images illustrating Ki67 staining (A) and the quantification of Ki67 positive cell proportion per unit area (B) on day 3.n=6, *p<0.05, ****p<0.0001. All data are presented as the mean ± SD.


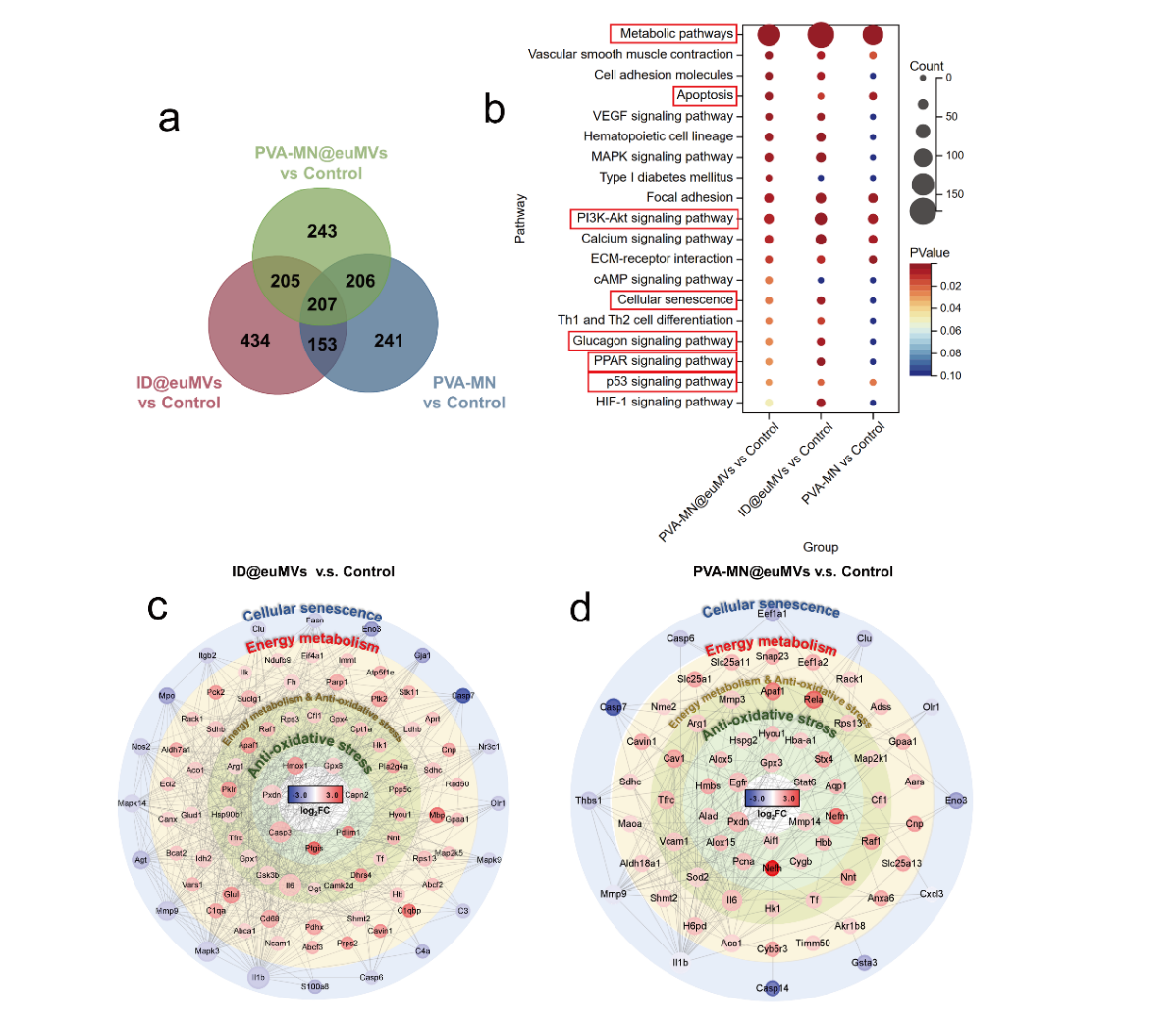


**Figure S8. Comparison of differentially expressed proteins revealed the therapeutic mechanism of the PVA-MN@euMVs in the diabetic pressure sore rat model.**

(A) Venn diagram comparing the total proteome in Control, PVA-MN, ID@euMVs and PVA-MN@euMVs groups. (B) Kyoto Encyclopedia of Genes and Genomes (KEGG) pathways analysis of DEPs of the PVA-MN, ID@euMVs and PVA-MN@euMVs compared with the Control group. (C) Protein‒protein interaction network of DEPs between ID@euMVs and Control. (D) Protein‒protein interaction network of DEPs between PVA-MN@euMVs and Control. n=3.


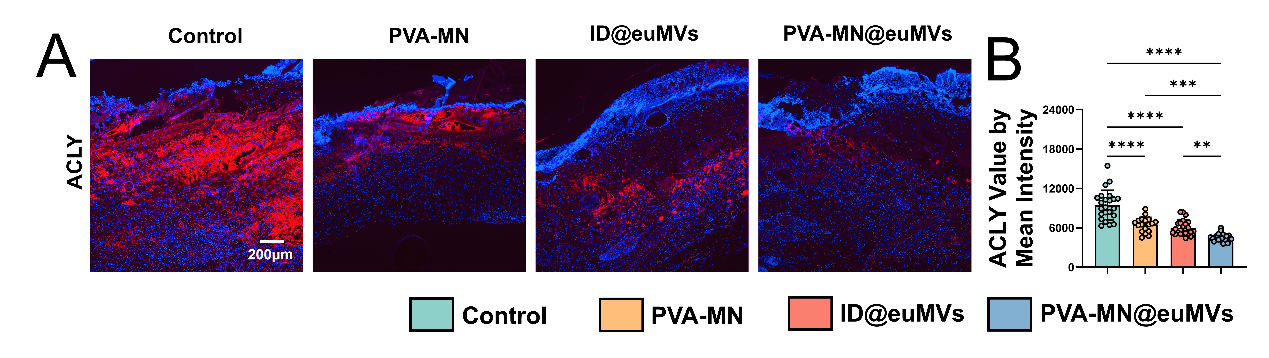


**Figure S9. ACLY expression in diabetic wounds on day 7.**

(A) Representative immunofluorescence images showing ACLY expression. (B) The average fluorescence intensity of ACLY analyzed using ImageJ. n=6, **p＜0.01, ***p < 0.001, ****p<0.0001. All data are presented as the mean ± SD.
